# SETJiP: Spatial and Extra Temporal Jigsaw Puzzles for Video Anomaly Detection †

**DOI:** 10.3390/s26092889

**Published:** 2026-05-05

**Authors:** Liheng Shen, Tetsu Matsukawa, Einoshin Suzuki

**Affiliations:** 1 Department of Information Science and Technology, Graduate School of Information Science and Electrical Engineering (ISEE), Kyushu University, Ito Campus, 744 Motooka, Nishi-ku, Fukuoka 819-0395, Japan; 2 Department of Informatics, Faculty of Information Science and Electrical Engineering (ISEE), Kyushu University, Ito Campus, 744 Motooka, Nishi-ku, Fukuoka 819-0395, Japan; matsukawa@inf.kyushu-u.ac.jp (T.M.); suzuki@inf.kyushu-u.ac.jp (E.S.)

**Keywords:** video anomaly detection, self-supervised learning, spatio-temporal representation learning, temporal jigsaw puzzles

## Abstract

Video Anomaly Detection (VAD) is commonly formulated as a one-class classification task. Global motion, with temporal variations across most pixels within an object-centric region, e.g., walking, is typically regular, whereas localized motion, e.g., waving, can be ambiguous. Decoupled spatial and temporal jigsaw puzzles (DSTJiP) is a self-supervised method that learns discriminative representations by predicting the original order of spatially and temporally shuffled patches. However, DSTJiP’s uniform sampling and equal weighting do not assign stronger supervision to global-motion examples within the temporal objective. Consequently, the temporal supervision allocated to global-motion examples may become insufficient across training-data regimes with varying proportions of these examples, deteriorating VAD performance. Nevertheless, excessively strengthening such supervision also degrades performance. To address these issues, we propose spatial and extra temporal jigsaw puzzles (SETJiP) with two RGB-only training schemes that provide stronger and more conservative temporal supervision for global-motion examples, respectively. One scheme strengthens temporal supervision on these examples via additional temporal jigsaw puzzles. The other does so more conservatively by upweighting their temporal jigsaw puzzles. Experiments on four VAD benchmarks show that both schemes improve on DSTJiP and remain highly competitive with state-of-the-art methods.

## 1. Introduction

Video Anomaly Detection (VAD) identifies unexpected events in video sequences, including objects, actions, and their interactions [1,2,3]. VAD is critical for real-world applications, such as automated surveillance [4,5,6], as it reduces the necessity for labor-intensive and time-consuming manual monitoring. Since the collection of a comprehensive set of anomalous data is often infeasible [7,8], VAD is commonly formulated as a one-class classification task. Specifically, a classifier is trained to learn the representations of regular events using only normal, unlabeled training examples [3,8,9], and this classifier is then employed to detect anomalies in test data. VAD is challenging, as it requires capturing highly discriminative representations to distinguish fine-grained motion-related anomalies from diverse normal examples, e.g., discriminating a running person from walking pedestrians within complex scenarios.

To detect fine-grained motion-related anomalies, capturing motion patterns is essential. However, diverse motion in training data is not equally informative and significant under the One-Class Video Anomaly Detection (OC-VAD) setting. Thus, we distinguish between global motion, characterized by temporal variations across a large proportion of pixels within an object-centric region, and localized motion confined to small subregions. In practice, global motion (e.g., walking) generally conveys more information and exhibits greater regularity, whereas localized motion (e.g., waving an arm) can be trivial and temporally ambiguous and can potentially lead to confusion with actual anomalies (e.g., throwing an object). Consequently, global motion usually plays a more critical role during training, suggesting that allocating greater temporal prioritization to global-motion examples can be beneficial for strengthening the discriminative power of representations. Here, temporal prioritization of global-motion examples refers to assigning them stronger supervision than other training examples within the temporal objective, e.g., by constructing more training instances from them and assigning them greater relative emphasis during loss optimization. Conversely, treating different motion examples uniformly can distract the model with trivial localized motion, thereby weakening its ability to model dominant normal motion patterns.

Under the OC-VAD setting, existing methods primarily leverage self-supervised learning (SSL) to learn spatio-temporal representations, focusing on improving training objectives to enhance the discriminability of the learned representations. Representative categories include generation-based and context-based methods [10,11,12]. Generation-based methods learn representations through fidelity-based objectives, e.g., reconstruction [13,14,15,16,17], prediction [18,19,20,21,22,23], and denoising [24,25,26,27]; however, they are susceptible to low-level appearance details, e.g., colors and textures, which hinder the learning of highly discriminative spatio-temporal representations. Context-based methods learn representations from discrete labels generated by data transformations, typically using context-based classification objectives, e.g., jigsaw puzzles [3,28], patch interval prediction [29], and rotation angle classification [30]; they can alleviate susceptibility to low-level features. Among these, jigsaw-puzzle-based methods are particularly promising, as their challenging objectives provide fine-grained supervision; for instance, predicting temporal labels for each shuffled patch encourages the model to encode fine-grained motion progression, e.g., the sequential dynamics of a walking person. As a representative method, decoupled spatial and temporal jigsaw puzzles (DSTJiP) [3] is highly attractive due to its minor data overhead, relying solely on RGB data without requiring extra modalities or synthetic data, thus avoiding the associated burdens of data extraction and preprocessing.

DSTJiP [3] learns representations by predicting the original order of spatially and temporally shuffled patches and jointly optimizing spatial and temporal objectives. It captures motion patterns through its temporal objective, which involves optimizing a loss term for temporal jigsaw puzzles. However, DSTJiP uniformly samples training examples and assigns them equal weights in the temporal loss. Consequently, it lacks an explicit mechanism to ensure sufficient temporal prioritization of global-motion examples across different training-data regimes in which such examples are relatively scarce or abundant. When these examples are relatively scarce during training, their low frequency can lead to insufficient temporal prioritization. This insufficiency biases the learned representations toward localized motion and hinders the detection of fine-grained motion-related anomalies, e.g., localized abnormal motion (A representative example of localized abnormal motion is a person who throws a bag while walking [31]: the act of throwing constitutes the localized abnormal motion, whereas the concurrent walking represents the normal global motion), under the one-class classification setting. Even when global-motion examples are abundant, the temporal prioritization allocated to them can still be mildly insufficient, because uniform sampling and equal weighting do not prevent it from being diluted by other training examples and the jointly optimized spatial objective. Nevertheless, excessively strengthening the temporal prioritization of these examples would bias learning toward global-motion patterns and reduce sensitivity to localized abnormal motion.

**Figure 1:**
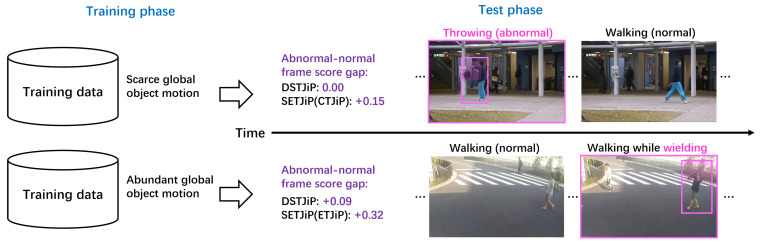
Motivating examples for SETJiP. Abnormal frames, objects, and regions are highlighted in pink. Wielding and throwing involve localized abnormal motion. A larger abnormal–normal frame score gap indicates better separability; scores are normalized to [0, 1]. Through CTJiP and ETJiP, SETJiP improves abnormal–normal separability in examples containing localized abnormal motion compared with DSTJiP.

To address these issues, we build upon DSTJiP to propose spatial and extra temporal jigsaw puzzles (SETJiP), which include two predefined training schemes: complementary temporal jigsaw puzzles (CTJiP) and emphasized temporal jigsaw puzzles (ETJiP). CTJiP provides a stronger form of temporal prioritization of global-motion examples by constructing additional temporal jigsaw puzzles and assigning an extra temporal loss term to them. ETJiP provides a more conservative form of temporal prioritization by assigning an extra loss to a subset of temporal jigsaw puzzles corresponding to global-motion examples while keeping the total temporal-loss weight unchanged, thereby increasing their relative temporal emphasis in a bounded manner. CTJiP and ETJiP are two temporal-prioritization schemes with different strengths. They are motivated by training-data regimes in which global-motion examples are relatively scarce and abundant, respectively, but are not intended to impose strict applicability boundaries. SETJiP inherits the RGB-only nature of DSTJiP without requiring additional modalities, synthetic data, increased model capacity, or extra inference cost. Figure 1 illustrates motivating examples in which SETJiP shows clearer abnormal–normal separation than DSTJiP for cases containing localized abnormal motion.

Our contributions are summarized as follows:

- We propose spatial and extra temporal jigsaw puzzles (SETJiP), which extends DSTJiP [3] by incorporating two decoupled temporal prioritization schemes. These schemes explicitly strengthen temporal prioritization of global-motion examples while preserving the RGB-only nature of the original framework.
- We introduce two training schemes, namely complementary temporal jigsaw puzzles (CTJiP) and emphasized temporal jigsaw puzzles (ETJiP), which provide stronger and more conservative temporal prioritization of global-motion examples, respectively. These designs are motivated by different training-data regimes rather than strict applicability boundaries.
- We comprehensively evaluate CTJiP and ETJiP on four public VAD benchmarks. They achieve leading or near-leading results among context-based SSL methods and remain highly competitive with methods from other categories, while preserving the RGB-only setting without extra modalities, synthetic data, or additional inference cost.

## 2. Related Work

Based on [10,11,12], self-supervised VAD methods can be mainly categorized into generation-based (SSL-Gen), contrastive-learning-based (SSL-Con), context-based (SSL-Ctt), and multi-pretext-task-based (SSL-Mul) methods.

### 2.1. Generation-Based VAD Methods

Generation-based VAD methods learn normality by recovering visual content under fidelity-driven objectives. They can be broadly divided into reconstruction-based and prediction-based branches.

In the reconstruction branch, some methods train autoencoders or recurrent generators to reconstruct input frames or short video clips and then derive anomaly scores from the reconstruction results, where larger reconstruction errors indicate higher anomaly likelihood [14,32]. Recent diffusion-based methods also learn normality through denoising-based generation and regard larger denoising or reconstruction discrepancies as abnormal evidence [24,26,33,34]. These methods expect the generator to capture dominant appearance regularities of normal events so that abnormal regions cannot be faithfully generated. However, pixel-level reconstruction errors can be strongly affected by low-level appearance deviations, such as illumination changes, texture or color shifts, and background clutter; moreover, expressive generators may partially reconstruct abnormal patterns.

In the prediction branch, some methods formulate VAD as future prediction [18] or missing-content completion [7], where the model learns temporal regularities of normal dynamics and identifies anomalies by larger prediction or completion errors. Subsequent studies improve temporal modeling through multi-scale aggregation [35] and prediction-consistency objectives such as skip-frame and bidirectional prediction [23]. Nevertheless, these methods still mainly depend on pixel-level prediction or completion errors, and their effectiveness can degrade when normal dynamics are highly diverse.

To alleviate the limitations of a single generator, some methods further integrate reconstruction and prediction within one framework [36,37], although such hybrid formulations may simultaneously inherit the weaknesses of both branches. Recent studies further combine predictive coding or frame prediction with reconstruction and memory mechanisms. For example, Hu et al. [38] combined next-frame prediction with predicted-frame reconstruction and introduced dynamic memory modules into the reconstruction network so that RGB-error-guided multiscale predictive coding can better emphasize stable normal cues while suppressing noisy variations. Within generation-based pipelines, some studies further introduce memory- or prototype-based mechanisms to store representative normal patterns for appearance and motion, thereby mitigating the encoding of trivial cues [5,8,13,15,39]. For example, Liu et al. [40] proposed a Stochastic Video Normality (SVN) network, which learns local appearance patterns in a deterministic manner while modeling global motion patterns as a stochastic distribution to capture their inherent uncertainty. It further couples appearance and motion with a mask autoencoder. Liu et al. [41] proposed a framework that uses external memories to record prototype features and enhances appearance-motion modeling with spatial–temporal fusion, multiscale spatial context, and temporal attention. Wang et al. [42] proposed a learnable memory-based framework that inserts a transformer-based memory module into the latent space of CNN-based autoencoders so that normal patterns can be modeled through learnable attention-based interactions instead of only through fixed similarity matching. Overall, generation-based methods and their memory-augmented extensions remain susceptible to low-level appearance variations and the intrinsic limitations of pixel-space generation.

### 2.2. Contrastive-Learning-Based VAD Methods

Contrastive-learning-based VAD methods learn representations by pulling together positive pairs and pushing apart negative pairs, thereby encouraging invariance to nuisance factors, such as illumination or background changes, while preserving discriminative semantics. However, representative contrastive-learning-based methods for VAD are relatively limited.

Representative methods include Cluster Attention Contrast (CAC) [43] and Hierarchical Semantic Contrast (HSC) [44]. CAC forms snippet-level inputs from videos, applies augmentation-based instance discrimination, and uses a cluster-attention mechanism to emphasize the normal subcategories most relevant to each sample, thereby improving the separability among different normal patterns [43]. HSC incorporates foreground-object and background-scene features with high-level semantics via video parsing and performs hierarchical contrast at the scene and object levels to capture scene-aware spatial and temporal context in complex scenes [44].

Overall, contrastive SSL can reduce the sensitivity of fidelity-driven objectives to low-level appearance deviations. However, its effectiveness often depends on carefully designed augmentation schemes for defining informative positive pairs, as well as auxiliary semantic pipelines, e.g., parsing or tracking, whose errors can propagate to representation learning and increase both training complexity and sensitivity to external component quality.

### 2.3. Context-Based VAD Methods

Context-based VAD methods commonly apply predefined transformations to input data to generate discrete pseudo-labels, and then train a classifier to predict them. Representative methods mainly differ in the form of discrete pseudo labels they predict, such as jigsaw permutations, rotation-related labels, or temporal interval classes.

A representative jigsaw-puzzle-based method is decoupled spatial and temporal jigsaw puzzles (DSTJiP) [3], which learns spatio-temporal representations by predicting the permutation index of either a spatial jigsaw, i.e., shuffling sub-patches within a frame, or a temporal jigsaw, i.e., shuffling patches across time, constructed from object-centric clips. As an RGB-only jigsaw-puzzle-based framework, DSTJiP provides fine-grained supervision for spatio-temporal representation learning. Nevertheless, the temporal learning framework in DSTJiP is prone to inadequately capturing motion patterns essential for VAD. Built upon DSTJiP, Shen et al. [28] proposed spatial and augmented temporal jigsaw puzzles (SATJiP), which introduces additional masked temporal jigsaw puzzles, constructed from global-motion examples, in which non-motion parts are randomly masked. However, introducing these jigsaw puzzles can risk overfitting to motion-relevant content. Furthermore, the masking scheme inadvertently introduces spurious temporal cues, such as variations in mask shape and location.

As a jigsaw-puzzle-based method, SETJiP differs from prior methods in that it prioritizes global-motion examples through its temporal objective, without relying on masking-based input modification for temporal learning. Existing methods mainly differ in how they construct temporal supervision. DSTJiP treats all training samples uniformly within the temporal objective and does not explicitly emphasize global-motion examples. SATJiP strengthens temporal supervision by constructing additional masked temporal jigsaw puzzles from global-motion examples; however, this masking scheme relies on input modification, which may introduce mask-induced spurious temporal cues. In contrast, SETJiP strengthens temporal learning without introducing masked samples. CTJiP constructs additional temporal jigsaw puzzles from selected global-motion examples, while ETJiP further avoids additional temporal jigsaw puzzle construction by reusing existing temporal jigsaw puzzles corresponding to global-motion examples and reallocating the temporal-loss weights under a fixed total temporal-loss scale. This design offers two advantages. First, by prioritizing global-motion examples at the objective level, SETJiP strengthens the learning of motion patterns that are more informative for anomaly detection. Second, by avoiding masking-based augmentation, it reduces the risk that the model relies on spurious temporal cues induced by the mask.

Other context-based methods formulate different discrete prediction tasks. Yang et al. [30] create appearance-based pseudo-anomalous samples by applying rotation operations to image patches and formulate a rotation-type prediction task. They further scramble the order of short frame triplets and formulate a four-category triplet classification task, e.g., non-anomaly, left-anomaly, right-anomaly, and total-anomaly, to encourage the model to recognize diverse local temporal irregularities. However, short triplets mainly capture local temporal order and provide limited temporal context, which can restrict their ability to model long-range action evolution and make them more sensitive to incidental appearance variations. Liu et al. [29] sample frames around a fixed middle frame and formulate a time-interval prediction task with discretized interval classes relative to the center, encouraging sensitivity to temporal interval and ordering. They further introduce a patch-level noise classification task by applying different noise levels to patches and predicting the corresponding noise level, thereby strengthening local appearance sensitivity. However, discretized frame intervals can be ambiguous in practice, since visually similar actions may correspond to different time intervals under varying motion speeds, which can deteriorate the learned representations.

Overall, context-based methods benefit from discrete supervisory signals that are less directly tied to pixel fidelity, but their effectiveness depends strongly on whether the designed pseudo labels are well aligned with the motion patterns relevant to anomaly detection.

### 2.4. Multi-Pretext-Task-Based VAD Methods

Multi-pretext-task-based VAD methods combine multiple objectives, e.g., reconstruction/prediction objectives and context-based classification tasks, to exploit their complementary advantages.

Georgescu et al. [45] formulated an early multi-pretext-task framework in which a shared backbone is trained with several pretext tasks, including temporal direction reasoning, motion irregularity discrimination, and object-appearance reconstruction, and task-wise discrepancies are aggregated for anomaly scoring. Building upon this paradigm, SSMTL++ [46] further expands the task set by introducing additional objectives such as jigsaw prediction and inpainting-style completion to improve robustness. Baradaran et al. [47] proposed an attention-based multi-task framework with complementary proxy tasks for appearance and motion modeling, including future semantic-segmentation prediction and motion-magnitude estimation guided by optical-flow magnitude. Zhang et al. [48] proposed a multi-scale framework that jointly optimizes three continuity-based proxy tasks using continuous and discontinuous clips. Specifically, it learns long-range temporal continuity for coarse motion patterns, discontinuity localization for fine local changes, and feature-space missing-frame estimation, where triplet loss is used to model motion relations and contrastive loss is used to model appearance relations across scenes.

While multi-pretext-task learning can alleviate the limitations of any single proxy task, it commonly faces two issues. First, different objectives can be imperfectly aligned and may introduce optimization difficulties, making joint training sensitive to task balancing and sometimes leading to suboptimal trade-offs for anomaly detection [49]. Second, it usually incurs higher engineering and computation costs, especially when proxy labels rely on off-the-shelf teachers, e.g., detectors and extractors, whose errors can propagate and increase sensitivity to external component quality.

## 3. Problem Formulation: One-Class Frame-Level Video Anomaly Detection

Under the one-class VAD setting, the model is trained using only normal examples. A common assumption is that the learned model generalizes better to unseen normal events than to abnormal events, so abnormal events tend to yield larger deviations and higher abnormality scores.

Let us denote the test dataset as $D^{test}=\left\{V_{k}|k\;=\;1,\ldots ,K\right\}$, where $V_{k}$ represents the *k*-th video, and *K* is the number of test videos. We further denote $V_{k}$ as $V_{k}\;=\;\left\{I_{i}^{k}|i\;=\;1,\ldots ,T^{k}\right\}$, where $I_{i}^{k}$ represents the *i*-th frame, and $T^{k}$ is the number of frames in $V_{k}$. $I_{i}^{k}$ and its temporally adjacent frames are stacked to construct a mini-clip $v_{i}^{k}$ with length *l* ($l\ll T^{k}$).

We define the ground-truth label of $I_{i}^{k}$ as $Y\left(I_{i}^{k}\right)\in \left\{0,1\right\}$, where 0 and 1, respectively, refer to normal and abnormal frames. The predicted (frame-level) abnormality score of $I_{i}^{k}$ is denoted as ${\tilde{S}}_{i}^{k}\in \left[0,1\right]$, which corresponds to the model’s confidence in classifying the frame as abnormal. The predicted label is obtained by thresholding $\hat{Y}\left(I_{i}^{k}\right)\;=\;I\left[{\tilde{S}}_{i}^{k}>\tau \right]$, where τ is a decision threshold.

**Figure 2:**
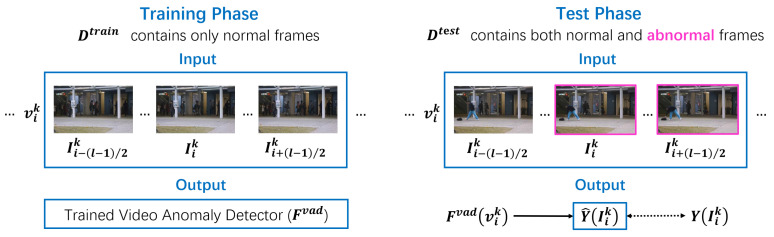
Overview of One-Class Frame-Level Video Anomaly Detection. Notations correspond to those explained in Section 3.

The objective of frame-level VAD is to train a model $F^{\mathrm{vad}}$ using a training dataset $D^{\mathrm{train}}$, whose structure is the same as that of $D^{\mathrm{test}}$, such that the model predicts $F^{\mathrm{vad}}\left(v_{i}^{k}\right)\;=\;{\tilde{S}}_{i}^{k}$ for each center frame $I_{i}^{k}$ and thus $\hat{Y}\left(I_{i}^{k}\right)$ matches the ground-truth label $Y(I_{i}^{k})$. We follow the paradigm of one-class anomaly detection, where $D^{\mathrm{train}}$ contains only normal examples, whereas $D^{\mathrm{test}}$ includes both normal and abnormal examples. Figure 2 shows an overview of One-Class Frame-Level Video Anomaly Detection.

## 4. Proposed Method: SETJiP

**Figure 3:**
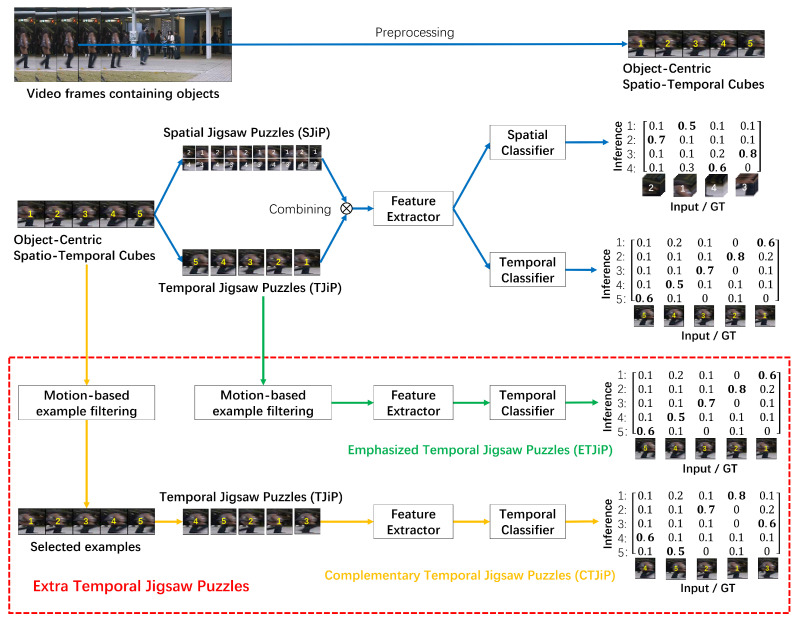
Overview of SETJiP framework, an extension of DSTJiP [3]. The extra temporal jigsaw puzzles (red dotted bounding box) involve two decoupled schemes: complementary temporal jigsaw puzzles (CTJiP) and emphasized temporal jigsaw puzzles (ETJiP). Blue arrows denote the original DSTJiP procedure, while green and orange arrows indicate the procedures of ETJiP and CTJiP, respectively. Feature extractors and temporal classifiers share the same model parameters, respectively. During the test phase, SETJiP follows the same procedure as DSTJiP [3]. Note that the ETJiP branch is drawn in an equivalent illustrative form for better comprehensibility.

Figure 3 illustrates the overall architecture of the proposed spatial and extra temporal jigsaw puzzles (SETJiP). Built upon DSTJiP [3], SETJiP is designed to address insufficient and excessive temporal prioritization of global-motion examples under training-data regimes with varying proportions of global-motion examples. Specifically, SETJiP introduces two decoupled schemes, CTJiP and ETJiP, which provide stronger and more conservative temporal prioritization of global-motion examples, respectively. CTJiP achieves this by constructing additional temporal jigsaw puzzles and assigning an extra subset-specific temporal loss, whereas ETJiP does so more conservatively by assigning an extra temporal loss exclusively to temporal jigsaw puzzles corresponding to global-motion examples while keeping the total temporal-loss weight unchanged, thereby increasing their relative emphasis within the temporal objective in a bounded manner.

During inference, SETJiP follows the same testing procedure as DSTJiP [3] (detailed in Algorithm 1) and therefore introduces no additional computational overhead. In the remainder of this section, we first review DSTJiP as the basis of our method and then present the details of SETJiP.

### 4.1. Base Method: DSTJiP

DSTJiP is a self-supervised method for Video Anomaly Detection that learns spatio-temporal representations by solving jigsaw puzzles. It builds object-centric spatio-temporal cubes by stacking patches cropped from temporally adjacent frames. From each cube, it constructs either spatial jigsaw puzzles (SJiP, shuffling uniformly partitioned mini-patches within each patch) or temporal jigsaw puzzles (TJiP, shuffling patches along the temporal dimension) and requires the model to predict the original indices. At test time, the model’s prediction confidence for the unshuffled state in both spatial and temporal puzzles is used to calculate frame-level abnormality scores.
**Algorithm 1 **DSTJiP test phase (SETJiP follows the same procedure)
 **Require:** 
Test videos $D^{test}\;=\;\left\{V_{k}|k\;=\;1,\ldots ,K\right\}$, $V_{k}\;=\;\left\{I_{i}^{k}|i\;=\;1,\ldots ,T^{k}\right\}$; trained solver J(·;θ); *l* (odd), (H×W), *n*; weight σ; decision threshold τ
 **Ensure:** 
Predicted label lists $\left[{\hat{Y}}^{1},\ldots ,{\hat{Y}}^{K}\right]$, where ${\hat{Y}}^{k}\;=\;\left[\hat{Y}\left(I_{1}^{k}\right),\ldots ,\hat{Y}\left(I_{T^{k}}^{k}\right)\right]$.// Preprocessing  1:$C_{\mathrm{all}}^{test}\leftarrow \mathrm{BUILDALLCUBES}\left(D^{test},l,H,W\right)$// Inference  2:**while** $C_{\mathrm{all}}^{test}$ is not empty **do**  3:   Sample a mini-batch of cubes $C\subset C_{\mathrm{all}}^{test}$ and remove them from $C_{\mathrm{all}}^{test}$   // Each cube will have a spatial and temporal object normality scores.  4:   **for** each cube $c_{i,m}^{k}\in C$ **do**  5:     // $c_{i,m}^{k}$ can be seen as unshuffled SJiP and TJiP  6:     $M_{i,m}^{s},\;M_{i,m}^{t}\leftarrow J\left(c_{i,m}^{k};\theta \right)$      // Process unshuffled SJiP and TJiP.  7:     $r_{i,m}^{s}\leftarrow \min\;\left(\mathrm{diag}(M_{i,m}^{s})\right)$     // Object-level spatial normality score, by Equation (4)  8:     $r_{i,m}^{t}\leftarrow \min\;\left(\mathrm{diag}(M_{i,m}^{t})\right)$     // Object-level temporal normality score, by Equation (4)  9:   **end for**10:**end while**11:**for** k = 1,…,K **do**12:   $i_{\min}\leftarrow \frac{l\;+\;1}{2},\;\;i_{\max}\leftarrow T^{k}-\frac{l-1}{2}$        // First, last valid center frame of cubes13:   $R_{1:T^{k}}^{k,s}\leftarrow 1,\;\;R_{1:T^{k}}^{k,t}\leftarrow 1$   // Initialize frame-level spatial and temporal normality scores.   // Boundary and no-object frame score filling: set as normal (normality score = 1).14:   **for** $i\;=\;i_{\min},\ldots ,i_{\max}$ **do**15:     **if** $M_{i}>0$ **then**16:        $R_{i}^{k,s}\leftarrow \min\left\{r_{i,1}^{s},\ldots ,r_{i,M_{i}}^{s}\right\},\;\;R_{i}^{k,t}\leftarrow \min\left\{r_{i,1}^{t},\ldots ,r_{i,M_{i}}^{t}\right\}$        // by Equation (5)17:     **end if**18:   **end for**   // min–max normalization, video-wise19:   ${\bar{R}}_{i_{\min}:i_{\max}}^{k,s}\leftarrow \mathrm{MINMAXNORM}\;\left(R_{i_{\min}:i_{\max}}^{k,s}\right)$20:   ${\bar{R}}_{i_{\min}:i_{\max}}^{k,t}\leftarrow \mathrm{MINMAXNORM}\;\left(R_{i_{\min}:i_{\max}}^{k,t}\right)$   // 1D Gaussian filtering, video-wise21:   ${\tilde{R}}_{1:T^{k}}^{k,s}\leftarrow \mathrm{SCORESMOOTHING}\left({\bar{R}}_{1:T^{k}}^{k,s}\right)$22:   ${\tilde{R}}_{1:T^{k}}^{k,t}\leftarrow \mathrm{SCORESMOOTHING}\left({\bar{R}}_{1:T^{k}}^{k,t}\right)$23:   **for** $i\;=\;1,\ldots ,T^{k}$ **do**24:     ${\tilde{S}}_{i}^{k}\leftarrow 1-\left(\left(1-\sigma \right){\tilde{R}}_{i}^{k,s}\;+\;\sigma {\tilde{R}}_{i}^{k,t}\right)$        // Reverse to abnormality score25:     $\hat{Y}\left(I_{i}^{k}\right)\leftarrow I\left[{\tilde{S}}_{i}^{k}>\tau \right]$         // 1: Abnormal; 0: Normal26:   **end for**27:**end for**28:**return** $\left[{\hat{Y}}^{1},\ldots ,{\hat{Y}}^{K}\right]$

#### 4.1.1. Preprocessing: Construction of Spatial and Temporal Jigsaw Puzzles

DSTJiP [3] employs a YOLOv3 detector [50] to extract all objects frame-by-frame. For each object detected in frame *i*, DSTJiP constructs an object-centric spatio-temporal cube c by simply stacking patches cropped from its temporally adjacent frames $\left\{i-\frac{l-1}{2}\ldots ,i-1,i,i\;+\;1,\ldots .,i\;+\;\frac{l-1}{2}\right\}$ using the associated bounding box $b^{i}$ of the object, assuming the cube length *l* is an odd number (detailed in Algorithm A1). The bounding box $b^{i}$ is represented as follows:

(1) $$b^{i}\;=\;\left(x_{1}^{i},y_{1}^{i},x_{2}^{i},y_{2}^{i}\right),$$

where $\left(x_{1}^{i},y_{1}^{i}\right)$ and $\left(x_{2}^{i},y_{2}^{i}\right)$ denote the horizontal and vertical coordinates of the top-left and bottom-right corners of the bounding box, respectively.

The object-centric spatio-temporal cube is denoted as $c\;=\;c_{1:l}$, where $c_{t}$ is a patch at temporal index *t* within the cube. DSTJiP resizes all the extracted patches into a fixed spatial size (H×W). For DSTJiP, c serves as the basic example and is either spatially or temporally shuffled to construct jigsaw puzzles.

**SJiP.** DSTJiP partitions $c_{t}$ into n×n equal-sized mini-patches and spatially shuffles them using a random permutation $P^{s}$ to construct spatial jigsaw puzzles (SJiP), as detailed in Algorithm A2. In SJiP, the shuffled mini-patches within each patch share the same spatial permutation. Thus, SJiP can be regarded as c being spatially shuffled by a single permutation.

**TJiP.** DSTJiP shuffles each patch in c in the temporal direction with a random permutation $P^{t}$ to construct temporal jigsaw puzzles (TJiP), as detailed in Algorithm A3. However, for c containing only static content, inferring the shuffled temporal order based on visual cues is ill-posed [3]. Therefore, DSTJiP does not shuffle such c temporally. To this end, DSTJiP determines whether c contains only static content by computing the maximum absolute difference $g_{m}\left(\cdot \right)$ between $c_{l}$ and $c_{1}$ in c. $g_{m}\left(c\right)$ is computed as follows:

(2) $$g_{m}\left(c\right)\;=\;\max(|c_{l}-c_{1}|).$$

Here, max(·) returns the largest element in a given tensor. DSTJiP applies temporal shuffling only to those c of which $g_{m}\left(c\right)$ exceeds a threshold δ. Otherwise, for those c of which $g_{m}\left(c\right)$ is below δ, DSTJiP preserves their original temporal sequence to generate TJiP, which has a unique fixed answer. Temporally unshuffled static content constitutes a specific form of a temporal pattern. It remains essential for model training, as anomaly detection is performed from both spatial and temporal views for all cubes.

For each c in the training phase, either spatial or temporal shuffling is performed, not both. In implementation, each sampled cube carries a fixed SJiP/TJiP assignment $f_{t}\left(c\right)\in \left\{0,1\right\}$, determined in the dataset loader by dataset-index parity, as detailed in Algorithms 2 and 3. SJiP and TJiP share the same size as c and contain explicit temporal structure.

#### 4.1.2. Training Phase: Self-Supervised Learning by Solving SJiP and TJiP

DSTJiP [3] constructs both SJiP and TJiP and trains a model employing a 3D CNN backbone to predict the index of each spatially shuffled mini-patch or each temporally shuffled patch in c. A mini-batch consists of two sets, $Q_{s}$ and $Q_{t}$, denoting the set of SJiP and TJiP samples, respectively. DSTJiP optimizes the model using the Cross-Entropy (CE) loss, though Mean Squared Error (MSE) loss can also be employed [3].

For a jigsaw puzzle *p*, the loss function is computed as follows:

(3) $$\begin{array}{c} L_{p}\;=\;\left\{\begin{array}{ccc} & L_{s}\;=\;\frac{1}{n^{2}}\sum_{j=1}^{n^{2}}\mathrm{CE}\left(s_{j},\hat{s_{j}}\right),\; & p\in Q_{s},\\ & L_{t}\;=\;\frac{1}{l}\sum_{i=1}^{l}\mathrm{CE}\left(t_{i},\hat{t_{i}}\right),\; & p\in Q_{t}, \end{array}\right. \end{array}$$

where $s_{j}$ and $t_{i}$ are the ground truth of spatial and temporal indexes, i.e., pseudo labels, respectively, while $\hat{s_{j}}$ and $\hat{t_{i}}$ are the inferred results.
**Algorithm 2 **SETJiP(CTJiP) training phase
 **Require:** 
Training videos $D^{train}\;=\;\left\{V_{k}|k\;=\;1,\ldots ,K\right\}$, $V_{k}=\left\{I_{i}^{k}|i\;=\;1,\ldots ,\;T^{k}\right\}$; cube length *l* (odd); resized patch size (H×W); grid size *n*; thresholds δ, μ; loss weights β, η; epochs *E*
 **Ensure:** 
Trained parameters θ// Preprocessing (same as DSTJiP)  1:$C_{\mathrm{all}}^{train}\leftarrow \mathrm{BUILDALLCUBES}\left(D^{train},l,H,W\right)$       // by Algorithm A1// Each sampled cube c carries a fixed SJiP/TJiP assignment.// Temporal-flag $f_{t}\left(c\right)\in \left\{0,1\right\}$, where $f_{t}\left(c\right)\;=\;0$: SJiP, $f_{t}\left(c\right)\;=\;1$: TJiP, determined by dataset index parity (assigned in the dataset loader).  2:Initialize jigsaw puzzle solver J(·;θ)←InitializeSolver()  3:**for** e = 1,…,E **do**  4:   $C_{\mathrm{pool}}^{train}\leftarrow C_{\mathrm{all}}^{train}$  5:   **while** $C_{\mathrm{pool}}^{train}$ is not empty **do**  6:     Sample a mini-batch of cubes $C\subset C_{\mathrm{pool}}^{train}$ and remove them from $C_{\mathrm{pool}}^{train}$     // Baseline decoupled SJiP and TJiP training (same as DSTJiP).     // With shuffled sampling, the spatial/temporal split ($f_{t}\left(c\right)\;=\;0/1$) is roughly balanced in expectation in a mini-batch.  7:     $C_{s}\leftarrow \left\{c\in C|f_{t}\left(c\right)\;=\;0\right\},\;\;C_{t}\leftarrow \left\{c\in C|f_{t}\left(c\right)\;=\;1\right\}$     // $C_{s}$, $C_{t}$ for constructing spatial, temporal jigsaw puzzles  8:     $Q_{s}\leftarrow \mathrm{CONSTRUCTSJIP}\left(C_{s},l,n\right)$          // by Algorithm A2  9:     $Q_{t}\leftarrow \mathrm{CONSTRUCTTJIP}\left(C_{t},l,\delta \right)$          // by Algorithm A310:     $L_{s}\leftarrow \mathrm{SPATIALLOSS}\left(J(Q_{s};\theta ),n\right)$          // by Equation (3)11:     $L_{t}\leftarrow \mathrm{TEMPORALLOSS}\left(J(Q_{t};\theta ),l\right)$          // by Equation (3)     // CTJiP: construct complementary temporal jigsaw puzzles from global-motion cubes (set $C_{m}$).12:     $C_{m}\leftarrow \left\{c\in C|g_{m}\left(c\right)>\delta \wedge g_{d}\left(c\right)>\mu \right\}$          // by Equations (2), (7) and (8)13:     $Q_{ct}\leftarrow \mathrm{CONSTRUCTCTJiP}\left(C_{m},l\right)$          // by Algorithm A414:     $L_{ct}\leftarrow \mathrm{COMPLEMENTARYTEMPORALLOSS}\left(J(Q_{ct};\theta ),l\right)$          // by Equation (9)// Final loss and parameter update15:     $L\leftarrow L_{s}\;+\;\beta L_{t}\;+\;\eta L_{ct}$          // Equation (11)     // set β + η>116:     $\theta \leftarrow \mathrm{UPDATE}\;\left(\theta ,\nabla_{\theta }L\right)$17:   **end while**18:**end for**19:**return** θ

#### 4.1.3. Test Phase: Video Anomaly Detection (VAD)

DSTJiP assumes that only the indices of the patches generated from normal examples can be accurately inferred, while those generated from abnormal examples cannot. Based on this assumption, in the test phase, the model processes unshuffled SJiP and TJiP and outputs probability matrices for the inferred spatial and temporal indices, where rows and columns correspond to the inferred indices and the input (the ground truth), respectively. We denote the *m*-th c corresponding to frame *i* as $c^{i,m}$ and the inferred spatial and temporal indices as $M_{s}^{i,m}$ and $M_{t}^{i,m}$. The spatial and temporal regularity scores of $c^{i,m}$, i.e., $r_{s}^{i,m}$ and $r_{t}^{i,m}$, are respectively defined as follows:

(4) $$\begin{array}{c} \left\{\begin{array}{c} r_{s}^{i,m}\;=\;\min\left(\mathrm{diag}\left(M_{s}^{i,m}\right)\right),\\ r_{t}^{i,m}\;=\;\min\left(\mathrm{diag}\left(M_{t}^{i,m}\right)\right), \end{array}\right. \end{array}$$

where diag(·) extracts the diagonal elements of a matrix as a vector, and min(·) returns the smallest element in a given vector. The regularity scores of the *i*-th frame, i.e., $R_{s}^{i}$ and $R_{t}^{i}$, are respectively defined as follows:

(5) $$\left\{\begin{array}{c} R_{s}^{i}\;=\;\min\left(r_{s}^{i,1},r_{s}^{i,2},\ldots ,r_{s}^{i,M_{i}}\right),\\ R_{t}^{i}\;=\;\min\left(r_{t}^{i,1},r_{t}^{i,2},\ldots ,r_{t}^{i,M_{i}}\right), \end{array}\right.$$

where $M_{i}$ denotes the total number of objects in the *i*-th frame.

The final abnormality score of the *i*-th frame, $S^{i}$, is computed as follows:

(6) $$\begin{array}{c} S^{i}\;=\;1-\left(\left(1-\sigma \right)R_{s}^{i}\;+\;\sigma R_{t}^{i}\right), \end{array}$$

where σ is the weight for $R_{t}^{i}$. DSTJiP applies a temporal 1D Gaussian filter to smooth the scores and performs max–min normalization [3]. The testing procedure is summarized in Algorithm 1.

### 4.2. Extra Temporal Jigsaw Puzzles

#### 4.2.1. Motivation

**Figure 4:**
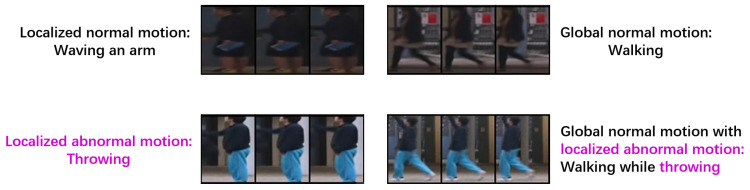
Illustrative examples for the motivation of SETJiP. Each panel shows three consecutive object-centric crops and the corresponding motion type. These examples illustrate the trade-off in temporal learning: too little emphasis on global motion can cause the temporal objective in jigsaw-puzzle-based self-supervised learning to focus overly on localized motion. In contrast, excessive emphasis may reduce the model’s sensitivity to localized abnormal motion occurring alongside global normal motion.

Under the one-class VAD setting, different motion patterns in training data are not equally informative for learning discriminative representations. In particular, global motion, characterized by temporal variations across a large proportion of pixels within an object-centric spatio-temporal cube, usually reflects dominant normal behavior and provides richer motion. By contrast, localized motion can sometimes be temporally ambiguous and may even resemble anomalies; for example, waving an arm may share local temporal variations with throwing an object (see Figure 4 for intuitive examples). Consequently, global-motion examples usually play a more critical role during training, and treating different motion patterns equally in temporal learning is suboptimal.

DSTJiP learns motion patterns through its temporal objective by predicting temporal jigsaw labels, while jointly optimizing this objective alongside the spatial objective. However, DSTJiP uniformly samples training examples and assigns them equal weights in the temporal loss. Consequently, it lacks an explicit mechanism to ensure sufficient temporal prioritization of global-motion examples across training-data regimes with varying proportions of global-motion examples. When global-motion examples are relatively scarce during training, their low frequency often leads to insufficient temporal prioritization for them, which biases the learned representations toward localized motion and weakens the modeling of dominant normal motion patterns. Even when global-motion examples are abundant, the temporal prioritization allocated to them can still be mildly insufficient, because uniform sampling and equal weighting do not prevent it from being diluted by other training examples and the jointly optimized spatial objective (A more detailed explanation of the motivation is provided in Appendix A.1, where this issue is further interpreted from a gradient-flow perspective).

On the other hand, overly strengthening temporal prioritization of global-motion examples is also undesirable. Excessive enhancement can bias learning too strongly toward global-motion patterns and reduce sensitivity to localized abnormal motion, such as when an anomaly occurs concurrently with global normal motion (e.g., walking while throwing in Figure 4). Therefore, temporal prioritization of global-motion examples should be strengthened in different degrees under different training-data regimes.
**Algorithm 3 **SETJiP(ETJiP) training phase
 **Require:** 
Training videos $D^{train}\;=\;\left\{V_{k}|k\;=\;1,\ldots ,K\right\}$, $V_{k}=\left\{I_{i}^{k}|i\;=\;1,\ldots \;,T^{k}\right\}$; cube length *l* (odd); resized patch size (H×W); grid size *n*; thresholds δ, μ; loss weights β, η; epochs *E*
 **Ensure:** 
Trained parameters θ// Preprocessing (same as DSTJiP)  1:$C_{\mathrm{all}}^{train}\leftarrow \mathrm{BUILDALLCUBES}\left(D^{train},l,H,W\right)$  // by Algorithm A1// Each sampled cube c carries a fixed SJiP/TJiP assignment.// Temporal-flag $f_{t}\left(c\right)\in \left\{0,1\right\}$, where $f_{t}\left(c\right)\;=\;0$: SJiP, $f_{t}\left(c\right)\;=\;1$: TJiP, determined by dataset index parity (assigned in the dataset loader).  2:Initialize jigsaw puzzle solver J(·;θ)←InitializeSolver()  3:**for** e = 1,…,E **do**  4:   $C_{\mathrm{pool}}^{train}\leftarrow C_{\mathrm{all}}^{train}$  5:   **while** $C_{\mathrm{pool}}^{train}$ is not empty **do**  6:     Sample a mini-batch of cubes $C\subset C_{\mathrm{pool}}^{train}$ and remove them from $C_{\mathrm{pool}}^{train}$     // Baseline decoupled SJiP and TJiP training (same as DSTJiP).     // With shuffled sampling, the spatial/temporal split ($f_{t}\left(c\right)\;=\;0/1$) is roughly balanced in expectation in a mini-batch.  7:     $C_{s}\leftarrow \left\{c\in C|f_{t}\left(c\right)\;=\;0\right\},\;\;C_{t}\leftarrow \left\{c\in C|f_{t}\left(c\right)\;=\;1\right\}$     // $C_{s}$, $C_{t}$ for constructing spatial, temporal jigsaw puzzles  8:     $Q_{s}\leftarrow \mathrm{CONSTRUCTSJiP}\left(C_{s},l,n\right)$   // by Algorithm A2  9:     $Q_{t}\leftarrow \mathrm{CONSTRUCTTJIP}\left(C_{t},l,\delta \right)$   // by Algorithm A310:     $L_{s}\leftarrow \mathrm{SPATIALLOSS}\left(J(Q_{s};\theta ),n\right)$   // by Equation (3)11:     $L_{t}\leftarrow \mathrm{TEMPORALLOSS}\left(J(Q_{t};\theta ),l\right)$   // by Equation (3)     // ETJiP: select a subset of temporal jigsaw puzzles originating from global-motion cube set $C_{m}$.12:     $C_{m}\leftarrow \left\{c\in C|g_{m}\left(c\right)>\delta \wedge g_{d}\left(c\right)>\mu \right\}$   // by Equations (2), (7) and (8)13:     $Q_{et}\leftarrow \mathrm{SELECTETJIP}\left(Q_{t},C_{m}\right)$   // by Algorithm A514:     $L_{et}\leftarrow \mathrm{EMPHASIZEDTEMPORALLOSS}\left(J\left(Q_{t};\theta \right),Q_{et},l\right)$   // by Equation (10)     // Final loss and parameter update15:     $L\leftarrow L_{s}\;+\;\beta L_{t}\;+\;\eta L_{et}$   // Equation (11)     // set β + η = 116:     $\theta \leftarrow \mathrm{UPDATE}\;\left(\theta ,\nabla_{\theta }L\right)$17:   **end while**18:**end for**19:**return** θ

#### 4.2.2. Overview

SETJiP incorporates two decoupled training schemes, complementary temporal jigsaw puzzles (CTJiP) and emphasized temporal jigsaw puzzles (ETJiP), which provide stronger and more conservative forms of temporal prioritization, respectively. Both CTJiP and ETJiP begin by selecting global-motion examples through motion-based example filtering. CTJiP then constructs additional temporal jigsaw puzzles for the selected examples and assigns an extra temporal loss to this subset. In contrast, ETJiP more conservatively assigns an extra temporal loss to temporal jigsaw puzzles corresponding to the selected examples while keeping the total temporal-loss weight unchanged. These two schemes are motivated by different training-data regimes rather than strict applicability boundaries. During inference, SETJiP follows the same procedure as DSTJiP and incurs no additional computational overhead.

#### 4.2.3. Motion-Based Example Filtering

Effective temporal representation learning relies on examples exhibiting meaningful temporal variations, particularly object-induced motion, rather than spurious temporal variations such as those caused by illumination changes. Therefore, we first filter out static object-centric cubes using the motion score $g_{m}\left(\cdot \right)$ defined in Equation (2), following DSTJiP [3]. Specifically, we retain only those object-centric cubes c where $g_{m}\left(c\right)$ exceeds a threshold δ. This manipulation effectively discards examples dominated by spurious variations.

Among the remaining cubes, we further select those exhibiting global motion, characterized by a substantial proportion of pixel changes between the first and last time steps. Following SATJiP [28], we quantify the degree of global motion $g_{d}\left(\cdot \right)$ as

(7) $$\begin{array}{c} g_{d}\left(c\right)\;=\;\frac{1}{HW}\sum_{h=1}^{H}\sum_{w=1}^{W}b(∥c_{l}^{h,w}-c_{1}^{h,w}∥), \end{array}$$

where $c_{t}^{h,w}$ denotes the pixel value at spatial position (h,w) in the patch $c_{t}$, and b(·) is a threshold-based binarization function that identifies changed pixels. We select those c where $g_{d}\left(c\right)$ exceeds a threshold μ.

The final subset $C_{m}$, containing only global-motion examples, is defined as follows:

(8) $$C_{m}:\;=\;\left\{\;c\in C|g_{m}\left(c\right)>\delta \;\wedge \;g_{d}\left(c\right)>\mu \;\right\}.$$

Here, δ inherits from DSTJiP [3], and $g_{m}\left(c\right)>\delta$ helps filter out examples with false global motion, e.g., those caused by illumonation changes.

#### 4.2.4. Complementary Temporal Jigsaw Puzzles (CTJiP)

**Definition and Procedure.** Complementary temporal jigsaw puzzles (CTJiP) provides a strong form of temporal prioritization of global-motion examples by constructing additional temporal jigsaw puzzles exclusively from the selected global-motion examples $C_{m}$ (detailed in Algorithm A4). Let $Q_{ct}$ denote the set of these additional TJiP instances. For a temporal jigsaw puzzle $p_{ct}\in Q_{ct}$, the complementary temporal loss $L_{ct}$ is defined as

(9) $$\begin{array}{cc} L_{ct} & =\frac{1}{l}\sum_{i=1}^{l}CE\left(t_{i},{\hat{t}}_{i}\right), \end{array}$$

where $t_{i}$ is the temporal index used as the pseudo-label and ${\hat{t}}_{i}$ is the predicted result. Since the original temporal-jigsaw loss is computed over all training examples, global-motion examples already participate in the original temporal objective. By introducing $L_{ct}$ on the selected global-motion examples, CTJiP makes these examples participate in both the all-example temporal loss and the additional subset-specific temporal loss, thereby explicitly strengthening their temporal prioritization. The training procedure of SETJiP(CTJiP) is summarized in Algorithm 2.

**Rationale.** CTJiP is designed to explicitly strengthen temporal prioritization of global-motion examples under the joint optimization of spatial and temporal objectives. This stronger enhancement is particularly well motivated when global-motion examples are relatively scarce during training. In such cases, their low frequency makes their temporal prioritization insufficient, while optimization may be dominated by other training examples and the spatial objective. CTJiP explicitly assigns an additional temporal loss term to global-motion examples. Moreover, by constructing additional temporal jigsaw puzzles exclusively from these examples, it increases the number of temporal jigsaw training instances derived from them within each mini-batch, thereby intensifying the supervision. This stronger and more explicit prioritization compensates for the insufficient temporal prioritization of these examples (More detailed explanations of CTJiP are provided in Appendix A.2, where this design is also interpreted from a gradient-flow perspective).

#### 4.2.5. Emphasized Temporal Jigsaw Puzzles (ETJiP)

**Definition and Procedure.** Emphasized temporal jigsaw puzzles (ETJiP) provides a conservative form of temporal prioritization of global-motion examples. Let $Q_{et}\subset Q_{t}$ denote the subset of temporal jigsaw puzzles whose source cubes belong to $C_{m}$. $Q_{et}$ can be obtained by selecting from $Q_{t}$ only those puzzles generated from cubes in $C_{m}$, as detailed in Algorithm A5. For a temporal jigsaw puzzle $p_{et}\in Q_{et}$, the emphasized temporal loss $L_{et}$ is defined as

(10) $$\begin{array}{cc} L_{et} & =\frac{1}{l}\sum_{i=1}^{l}CE\left(t_{i},{\hat{t}}_{i}\right), \end{array}$$

where $t_{i}$ is the temporal index used as the pseudo-label and ${\hat{t}}_{i}$ is the predicted result. The training procedure of SETJiP(ETJiP) is summarized in Algorithm 3.

Unlike CTJiP, which additionally constructs temporal jigsaw puzzles from the selected cubes and often introduces different temporal permutations, ETJiP does not construct additional temporal jigsaw puzzles and only reuses the already constructed TJiP instances in $Q_{t}$. Hence, the difference between $Q_{et}$ and $Q_{ct}$ lies in reuse versus additional construction, rather than in a subset relation between the two sets.

ETJiP introduces an additional subset-specific temporal loss term for global-motion examples while redistributing the original temporal-loss strength between the all-example temporal loss and the subset-specific temporal loss. As a result, ETJiP strengthens temporal prioritization of global-motion examples while keeping the overall temporal-loss scale unchanged.

**Rationale.** ETJiP is designed to conservatively enhance the temporal prioritization of global-motion examples under the joint optimization of spatial and temporal objectives. This design is particularly well motivated when global-motion examples are abundant during training. In such cases, strongly increasing their temporal prioritization can over-bias learning toward global-motion patterns and reduce sensitivity to localized abnormal motion. Therefore, rather than further increasing the overall temporal-loss scale, ETJiP prioritizes global-motion examples by reallocating temporal-loss strength toward the subset-specific temporal loss while reducing the relative emphasis on other examples within the temporal objective. Moreover, because ETJiP does not introduce additional temporal jigsaw puzzles or permutations, it avoids the additional implicit strengthening that would arise from increasing the number of temporal jigsaw training instances derived from these examples within each mini-batch. This conservative and bounded enhancement enables ETJiP to strengthen temporal prioritization of global-motion examples while limiting the risk of excessively biasing learning toward global-motion patterns (More detailed explanations of ETJiP are provided in Appendix A.3, where this design is also interpreted from a gradient-flow perspective).

### 4.3. Model Training: SETJiP(CTJiP) and SETJiP(ETJiP)

We adopt the DSTJiP [3] framework to process SJiP and TJiP. Additionally, we incorporate either CTJiP or ETJiP into the spatio-temporal learning [3] detailed in Section 4.1.2. The total loss function for SETJiP is formulated as:

(11) $$L\;=\;L_{s}\;+\;\beta L_{t}\;+\;\eta L_{x},$$

where $L_{s}$ and $L_{t}$ denote the losses for SJiP and TJiP (Equation (3)), respectively. The additional temporal loss $L_{x}$ corresponds to either $L_{ct}$ (Equation (9)) for CTJiP or $L_{et}$ (Equation (10)) for ETJiP. Finally, β and η serve as the weighting hyperparameters for $L_{t}$ and $L_{x}$ (i.e., $L_{ct}$ or $L_{et}$).

Based on the motivation of CTJiP, SETJiP(CTJiP) adopts a larger total temporal-loss weight than the spatial-loss weight (i.e., β + η>1), thereby providing stronger temporal prioritization of global-motion examples. By contrast, SETJiP(ETJiP) keeps the total temporal-loss weight unchanged relative to the spatial loss (i.e., β + η = 1), so that temporal prioritization of global-motion examples is strengthened more conservatively without increasing the overall temporal-loss scale. This design helps avoid excessively biasing learning toward global-motion patterns under joint spatio-temporal optimization.

The training procedures of SETJiP(CTJiP) and SETJiP(ETJiP) are summarized in Algorithms 2 and 3, respectively. During inference, both variants follow exactly the same procedure as DSTJiP [3]; details are given in Section 4.1.3 and summarized in Algorithm 1. Pseudocode for the subroutines is provided in Appendix A.5.

## 5. Experiments

### 5.1. Datasets

We conduct our experiments on four public benchmark datasets widely used in VAD research [3,7,8,18,45,51,52].

- **UCSD Ped1 (Ped1)** [53]: dataset comprises 34 training videos (6800 frames) and 36 test videos (7200 frames) with a resolution of 238×158. Abnormal events are defined as non-pedestrian entities (e.g., small cars, cyclists, and skaters) or anomalous pedestrian actions on the walkway.
- **UCSD Ped2 (Ped2)** [53]: dataset contains 16 training videos (2550 frames) and 12 test videos (2010 frames) with a resolution of 360×240. The definition of abnormal events aligns with that of Ped1.
- **CUHK Avenue (Avenue)** [31]: dataset consists of 16 training videos (15,328 frames) and 21 test videos (15,324 frames) at a resolution of 640×360. Normal examples include pedestrians walking, while abnormal examples include activities such as loitering, running, and throwing objects.
- **ShanghaiTech Campus (STC)** [54]: dataset includes 330 training videos (274,515 frames) and 107 test videos (42,883 frames) with a resolution of 856×480. The dataset covers object-related anomalies (e.g., motorcycles, bicycles, and cars) as well as abnormal human behaviors like robbery and fighting. STC is noted for its large scale and diverse scenarios and viewpoints.

**Figure 5:**
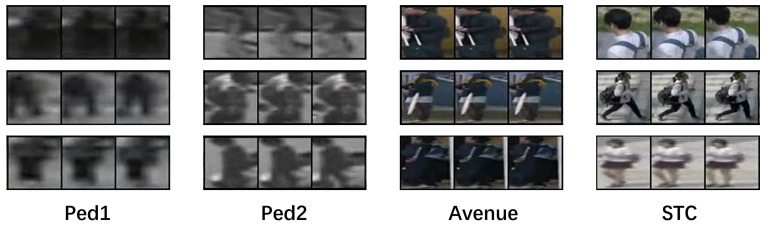
Visualized training examples. The four columns, from left to right, show examples from Ped1, Ped2, Avenue, and STC.

We show training examples generated from these datasets in Figure 5. For analyses involving localized abnormal motion, we use only Avenue and STC, as Ped1 and Ped2 do not contain such instances. Typical examples of localized abnormal motion include “throwing”, “pushing”, and “wielding”. There are 163 and 918 frames of localized abnormal motion in Avenue and STC, respectively, which are fewer than the total abnormal frames in Avenue (3712) and STC (17,326).

### 5.2. Evaluation Metric

In frame-level VAD, the abnormality score is defined such that a normal frame approaches 0, while an abnormal frame approaches 1. Following the majority of VAD methods [3,7,8,13,18,43,45,46,55,56], we employ the Area Under the Receiver Operating Characteristic Curve (AUROC) as our primary evaluation metric. Two common approaches exist for calculating AUROC in VAD. The first is micro-averaged AUROC [3,7,8,13,18,43,45,55], which concatenates all frames in a dataset to compute the overall frame-level AUROC. The second is macro-averaged AUROC [45,46,55,56], which calculates the frame-level AUROC for each video individually and averages these results. While many existing works [16,42,57,58] rely solely on micro-averaged AUROC to reflect overall performance, we report both micro- and macro-averaged AUROC to ensure comprehensive comparison with literature that utilizes both [45,46,55,56]. Additionally, we compute the Area Under the Precision–Recall Curve (AUPRC) to provide a more comprehensive assessment alongside AUROC. For all these metrics, higher values indicate better performance.

### 5.3. Implementation Details

**SETJiP.** Based on empirical observations, we set the batch size to 64, which stabilizes the optimization of multiple sub-losses. We set μ to 0 on Ped1 and Ped2 (This choice, which disables global-motion selection on Ped1 and Ped2, is motivated by the benchmark-level problem settings of these datasets. Ped1 and Ped2 mainly focus on anomalies involving the occurrence of uncommon moving objects or agents (e.g., bikers, skaters, small carts, or wheelchairs), rather than on localized motion irregularities within ordinary pedestrian motion. Therefore, the localized-motion-oriented motivation for global-motion selection is less directly matched to these benchmarks. This choice is made based on the benchmark’s official problem definition, rather than on prior knowledge of the specific anomaly instances appearing in the test data), and to 0.5 on Avenue and STC, following the characteristics of each dataset. The weight σ of $R_{t}$ is set to 0.6 to emphasize the contribution of the temporal view. For CTJiP, we set the loss weights β and η to 1 to prioritize temporal learning. For ETJiP, we set β and η to 0.9 and 0.1, respectively, to avoid overly emphasizing temporal learning.

The remaining hyperparameters were set to the same values as in DSTJiP [3]. For the jigsaw puzzle configuration, we set *H* and *W* to 64 and *n* to 3 for all datasets and set *l* to 9 for STC and 7 for Ped2 and Avenue. We set the threshold δ to 0.2 for filtering out examples with spurious motion. We train the network for 100 epochs on Avenue and STC and 50 epochs on Ped2. On Ped1, we adopt the settings used in Ped2, considering the similarity between the two datasets.

**Computation Cost.** We use an Intel(R) Core(TM) i9-9820X CPU and two NVIDIA RTX 3090 GPUs for training. Under the same training conditions, SETJiP requires approximately twice the model-training time of DSTJiP due to the additional optimization steps. Among the four datasets used in this study, Ped1 [53], Ped2 [53], Avenue [31], and STC [54], STC is the largest. On the STC dataset (274,515 training frames), the model-training time of SETJiP is less than 36 h. At test time, SETJiP follows the same inference procedure as DSTJiP. On the STC dataset (42,883 test frames), the inference time is less than 180 s. The reported model-training and inference times do not include offline preprocessing steps, such as object detection and object-centric cube construction.

**Baseline Methods.** We use the official codes and models of DSTJiP [3] (https://github.com/gdwang08/Jigsaw-VAD (accessed on 28 April 2026)) and SATJiP [28] to conduct our experiments. For the remaining baseline methods, we report the results as provided in the corresponding papers.

### 5.4. Comparison of the State-of-the-Art Methods

We compare SETJiP with state-of-the-art methods to examine its competitiveness. Since many representative methods do not consistently report results on all four benchmarks, Table 1 focuses on Ped2, Avenue, and STC, which are more commonly reported in prior work [3,28,29,30,44,49], so as to enable a broader and fairer cross-method comparison.

The empirical results in Table 1 show that, on STC, the strongest methods are mostly context-based. The heightened spatio-temporal complexity and illumination fluctuations in STC introduce substantial irrelevant low-level variations, which can more easily affect methods relying on fidelity-oriented objectives, thereby deteriorating the performance of SSL-Gen methods.

Although SETJiP (CTJiP) and SETJiP (ETJiP) are motivated by different training-data regimes, both achieve highly competitive performance compared with state-of-the-art VAD methods across the evaluated datasets. Moreover, SETJiP delivers leading or near-leading performance among context-based methods and remains highly competitive overall using only the RGB modality, thereby avoiding multi-modal integration or external data-related overhead (‡).

### 5.5. Comparison with the Most Relevant Methods

We further compare SETJiP (CTJiP) and SETJiP (ETJiP) with their most relevant baselines, DSTJiP [3] and SATJiP [28], on all four benchmarks with all three metrics to provide a more complete validation of the effectiveness of the proposed schemes. We evaluate both VAD performance and the ability to detect localized abnormal motion, as shown in Table 2 and Table 3, respectively.

**VAD.** The empirical results in Table 2 show that SETJiP(CTJiP) achieves the best performance on Ped1 and Avenue, while SETJiP(ETJiP) achieves the best performance on STC. Both variants improve upon DSTJiP on multiple datasets, demonstrating the effectiveness of the proposed temporal-prioritization schemes.

**Detecting localized abnormal motion.** The evaluation of the detection of localized abnormal motion is realized by restricting the analysis to normal frames and abnormal frames that contain such motion. Note that only Avenue and STC contain examples with localized abnormal motion. In Table 3, all methods yield low AUPRC scores, which can be attributed to the severe class imbalance, as localized abnormal frames account for only 1% and 2% of the Avenue and STC test sets, respectively. This suggests that discriminating localized abnormal motion from diverse normal motion remains challenging.

SETJiP(CTJiP) and SETJiP(ETJiP) achieve comparable performance with SATJiP while outperforming DSTJiP on Avenue. On STC, performance diverges, where SETJiP (ETJiP) achieves the best results, while SETJiP (CTJiP) brings no improvement upon DSTJiP. Overall, SETJiP outperforms DSTJiP in this challenging setting.

**Design motivation and scheme dependence.** The ratios of global-motion examples among the training examples are 38.7% and 97.2% on Avenue and STC, respectively. Although CTJiP and ETJiP were originally motivated by training-data regimes in which global-motion examples are relatively scarce and abundant, respectively, the empirical results do not suggest a strict applicability boundary between them. Specifically, although SETJiP(CTJiP) and SETJiP(ETJiP) show advantages on Avenue and STC, respectively, SETJiP(ETJiP) also performs well on Avenue. These results suggest that the key benefit of CTJiP and ETJiP lies in explicitly strengthening the temporal prioritization of global-motion examples. Therefore, the regime-based distinction is better viewed as a design motivation than as a hard rule for scheme selection.

### 5.6. Frame-Level VAD Examples

**Figure 6:**
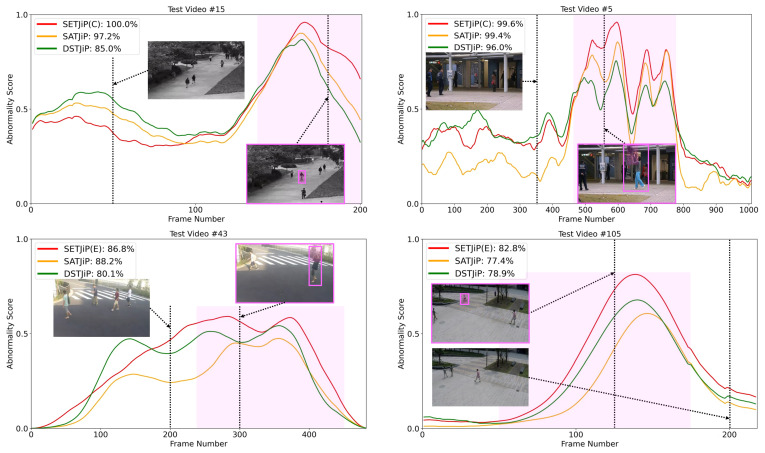
Frame-level VAD Examples of DSTJiP, SATJiP, and SETJiP. Abnormal frames, objects, and regions are highlighted in pink. VAD requires abnormality scores of abnormal frames to be higher than those of normal frames. Each method’s micro-averaged AUROC (%) value for each video clip is given. Examples are selected from Ped1, Avenue, and STC. We report results using SETJiP(CTJiP) for Ped1 and Avenue and SETJiP(ETJiP) for STC.

In Figure 6, we compare the performance of DSTJiP, SATJiP, and SETJiP by analyzing their frame-level abnormality score curves in selected video clips. While SATJiP effectively distinguishes between normal and abnormal frames, it tends to assign relatively low abnormality scores to abnormal frames compared with SETJiP, indicating a risk of overlooking anomalies. Although SETJiP sometimes assigns higher abnormality scores to normal frames than DSTJiP and SATJiP, it generally assigns even higher abnormality scores to abnormal frames, resulting in improved VAD performance.

### 5.7. Object-Level VAD Examples

**Figure 7:**
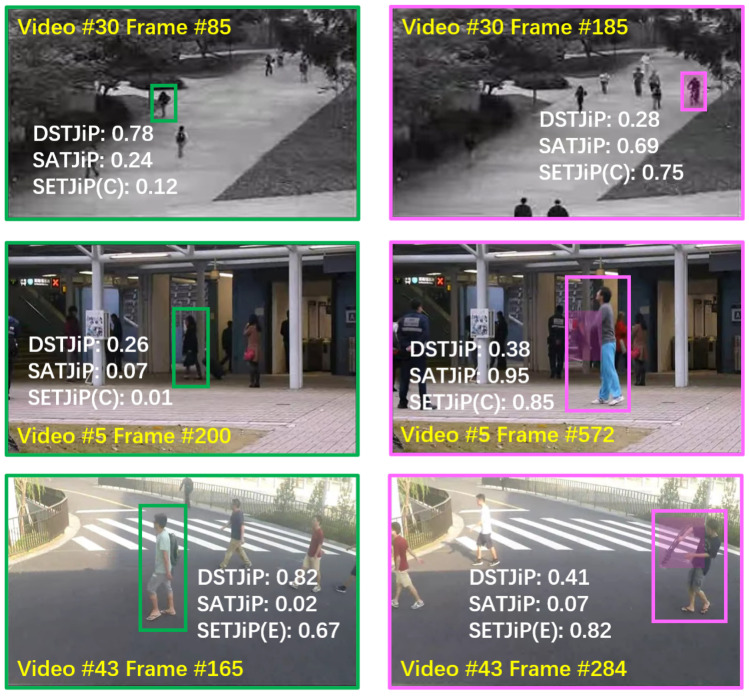
Object-level VAD examples of DSTJiP, SATJiP, and SETJiP. (**Left groups**): Normal object examples, highlighted in green. (**Right groups**): Abnormal object examples, highlighted in pink. For each object, the temporal abnormality scores derived from DSTJiP, SATJiP, and SETJiP are provided, as the temporal view is more relevant and direct than the spatial view for evaluating abnormal motion detection. Ideally, abnormality scores should be high for abnormal examples and low for normal examples. Examples are selected from Ped1, Avenue, and STC. We report results using SETJiP(CTJiP) for Ped1 and Avenue and SETJiP(ETJiP) for STC.

We investigate the characteristics of DSTJiP, SATJiP, and SETJiP through object-level examples. In Figure 7, we observe that, on Ped1, DSTJiP shows inferior performance on both normal and abnormal examples. On the other hand, SATJiP and SETJiP achieve better performance. On Avenue, both SETJiP and SATJiP perform well in detecting abnormal examples, whereas DSTJiP demonstrates suboptimal performance. On STC, SATJiP tends to assign low abnormality scores to abnormal examples. SETJiP exhibits clearer separation, assigning higher abnormality scores to abnormal examples.

### 5.8. Supplementary Experimental Results in Appendix

#### 5.8.1. Comparison of the Most Relevant Methods

Section Appendix B.1 offers further analysis of our proposed method in comparison with key baselines. As a supplement to Section 5.5, we examine temporal-view performance and the impact of the masking scheme [28].

**Temporal view.** The results indicate that the performance of the temporal view generally aligns with that of the spatio-temporal view, where SETJiP (CTJiP) performs best on Avenue while SETJiP (ETJiP) performs best on STC.

**Incorporating the masking scheme.** Empirical results indicate that incorporating the masking scheme [28] leads to mixed effects across datasets and does not provide consistent performance gains.

#### 5.8.2. Ablation Study

Section Appendix B.2 details ablation studies aimed at isolating the effects of individual design choices in CTJiP and ETJiP. We assess how different temporal learning mechanisms affect performance.

**Components in CTJiP and ETJiP.** We assess individual components by incrementally integrating temporal learning mechanisms and example selection schemes. Although the optimal configuration depends on the dataset, incorporating these components tends to improve performance over the base model.

**Prioritized and balanced temporal learning.** We compare balanced and temporally prioritized learning regimes. The results indicate method-dependent behavior: temporal prioritization can be beneficial for CTJiP on certain datasets, whereas ETJiP generally performs better with balanced learning.

#### 5.8.3. Parameter Analysis

Section Appendix B.3 details a parameter sensitivity analysis for CTJiP and ETJiP, focusing on hyperparameters that control temporal learning and example selection. We examine (i) temporal loss weight configurations: β and η and (ii) three scalar hyperparameters: δ and μ (motion-based filtering) and σ (abnormality score weighting).

**Temporal loss weights for CTJiP and ETJiP.** The results indicate that the effect of temporal loss weights differs substantially between CTJiP and ETJiP. CTJiP benefits from temporally prioritized configurations (β + η>1), particularly on Ped1 and Ped2, where emphasizing temporal learning leads to improved performance. In contrast, ETJiP achieves more stable performance under balanced or weakly prioritized settings (e.g., β + η = 1).

**Thresholds for motion-based example filtering and the weight for abnormality score.** The results indicate that the performance is reliable within a moderate range, δ∈[0.1,0.3], μ∈[0.3,0.5], and σ∈[0.5,0.7].

### 5.9. Limitations

**Practical applicability.** In this study, SETJiP is primarily intended for offline analysis of recorded videos. While our method achieved an inference speed of approximately 238 FPS (42,883 frames/180 s), the reported inference time does not include preprocessing steps, such as object detection using YOLOv3 and region extraction. These preprocessing steps are relatively lightweight and may be compatible with real-time processing in an appropriate system setting. However, validating real-time deployment under different hardware settings, streaming conditions, and system-level constraints is beyond the scope of the present work. Evaluating robustness and efficiency in real-world, unseen environments remains an important direction for future research.

**Motion-based filtering and cross-scene robustness.** SETJiP still has several limitations. First, it relies on motion-based filtering to select global-motion examples, so the thresholds δ and μ should be regarded as practical hyperparameters rather than universally fixed constants. Although the sensitivity analysis suggests reliable performance within moderate ranges, scene-specific motion characteristics may still affect which examples are selected as global-motion examples.

Second, this study follows the standard within-benchmark VAD evaluation protocol and does not evaluate adaptation to new surveillance settings with different cameras, scenes, viewpoints, illumination conditions, backgrounds, object scales, or normal motion patterns. Related studies in other visual inspection tasks, such as unsupervised domain adaptation for crack segmentation [65,66], suggest that improving robustness under such distribution shifts is an important future direction. More detailed discussion is provided in Appendix B.4.

## 6. Conclusions

In this paper, we propose SETJiP, a self-supervised RGB-only VAD method built upon DSTJiP. SETJiP introduces two decoupled training schemes, CTJiP and ETJiP, which provide stronger and more conservative temporal prioritization of global-motion examples, respectively. Experiments on four public VAD benchmark datasets demonstrate that both variants improve upon DSTJiP across multiple benchmarks and remain highly competitive with state-of-the-art methods while maintaining the RGB-only setting without requiring extra modalities, synthetic data, or additional inference cost.

Looking ahead, an interesting direction is to explore whether complementary modalities can further enhance Video Anomaly Detection. For example, skeleton data can provide explicit posture information, whereas optical flow can directly describe motion. In this sense, extending the spatial and temporal jigsaw objectives to non-RGB modalities may further improve the detection of abnormal human actions.

## Figures and Tables

**Table 1:** Comparison of the state-of-the-art methods in VAD on the commonly reported benchmarks. Performance is measured by micro-averaged AUROC (%). RGB image (**R**), optical flow (**F**), and human pose (**P**). **SSL-Gen:** Methods of generation-based SSL tasks. **SSL-Con:** Methods of contrastive-learning-based SSL tasks. **SSL-Ctt:** Methods of context-based SSL tasks. **SSL-Mul:** Methods of multi-pretext SSL tasks. *: Jigsaw-puzzle-based SSL methods. ‡: Methods that require additional data-related overhead (e.g., extra modality extraction, external datasets beyond the VAD benchmarks, or synthetic data generation). **Bold** figures: the best score. Underlined figures: the second-best score.

Category	Method	Modality	Ped2	Avenue	STC
SSL-Gen	BDPN ‡ [19]	R + F	98.3	90.3	78.1
	OGMR-Net ‡ [20]	R + F	97.4	92.6	74.9
	STM-AE ‡ [59]	R + F	98.1	89.8	73.8
	MAAM-VAD ‡ [15]	R + F	97.7	90.9	71.3
	BFI-VAD ‡ [21]	R + F	98.9	89.7	75.0
	MoCoDAD [60]	P	–	89.0	77.6
	DAST-Net [58]	R	97.9	89.8	73.7
	MLRA-VAS [16]	R	96.2	87.9	77.8
	AMP-Net [41]	R	98.7	92.4	78.8
	DHVAD [24]	R	98.4	91.0	77.5
	MAG-PDM [26]	R	98.6	91.3	79.2
	SDMAE ‡ [25]	R	95.4	91.3	79.1
	SSAE [61]	R	–	90.2	80.5
	U-net w TransMem [42]	R	98.1	88.5	72.5
	TrajREC [62]	P	–	89.4	77.9
	LGN-Net [22]	R	97.1	89.3	73.0
	MMAN-CMM [17]	R	99.0	92.1	79.3
	BiSP [23]	R	98.6	89.5	76.4
SSL-Con	CAC [43]	R	–	87.0	79.3
	HSC ‡ [44]	R + P	98.1	**93.7**	83.4
SSL-Mul	SS-MTL [45]	R	97.5	91.5	82.4
	SS-MTL-U ‡ [63]	R	–	93.0	83.7
	SSMTL++v1 ‡ [46]	R + F	–	**93.7**	82.9
	SSMTL++v2 ‡ [46]	R + F	–	91.6	83.8
	SLMPT [49]	R	97.6	90.9	78.8
	GiCiSAD [64]	P	–	89.6	78.0
SSL-Ctt	STPTL-VAD [30]	R	99.1	91.9	81.1
	TIPNC [29]	R	98.6	91.7	83.7
	DSTJiP * [3]	R	98.9	92.1	**84.2**
	SATJiP * ‡ [28]	R + F	**99.4**	93.2	84.1
	SETJiP(CTJiP) *	R	99.3	93.4	83.8
	SETJiP(ETJiP) *	R	98.8	93.3	84.1

**Table 2:** Comparison of the most relevant methods of SETJiP in VAD. Performance is measured by micro-averaged AUROC (%), macro-averaged AUROC (%), and AUPRC (%). Higher values indicate better performance. ★: best results between CTJiP (C) and ETJiP (E). **Bold** figures: the best score. Underlined figures: the second-best score.

Method	Micro-Averaged AUC				Macro-Averaged AUC				AUPRC			
	Ped1	Ped2	Ave.	STC	Ped1	Ped2	Ave.	STC	Ped1	Ped2	Ave.	STC
DSTJiP [3]	77.9	98.9	92.1	**84.2**	82.9	**99.9**	92.3	89.9	81.1	99.8	81.9	**82.4**
SATJiP [28]	79.2	**99.4**	93.2	84.1	**87.3**	**99.9**	91.4	89.6	82.4	**99.9**	79.0	82.1
SETJiP (C)	**80.8** ★	99.3 ★	**93.4** ★	83.8	**87.3** ★	**99.9** ★	**93.3** ★	89.3	**83.7** ★	99.8 ★	83.5	81.0
SETJiP (E)	79.8	98.8	93.3	84.1 ★	83.9	99.8	91.4	**91.0** ★	81.7	99.7	**84.3** ★	**82.4** ★

**Table 3:** Comparison in detecting localized abnormal motion. Performance is measured by micro-averaged AUROC (%) and AUPRC (%). Higher values indicate better performance. ★: best results between CTJiP (C) and ETJiP (E). **Bold** figures: the best score. Underlined figures: the second-best score.

Method	Micro-Averaged AUROC		AUPRC	
	Avenue	STC	Avenue	STC
DSTJiP [3]	86.2	85.7	12.6	15.1
SATJiP [28]	**95.6**	88.6	21.4	17.0
SETJiP (CTJiP)	92.7	84.1	**24.8** ★	17.1
SETJiP (ETJiP)	94.6 ★	**88.7** ★	21.3	**21.6** ★

**Figure A1:**
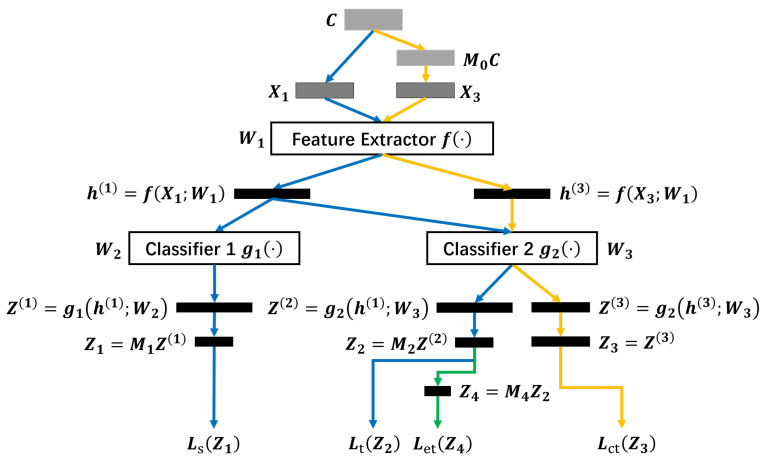
Schematic overview of the SETJiP framework from a gradient-flow perspective. Blue arrows denote the base DSTJiP procedure, orange arrows denote CTJiP, and green arrows denote ETJiP. CTJiP constructs additional temporal jigsaw puzzles from selected global-motion examples, whereas ETJiP reuses a subset of existing temporal jigsaw puzzles and introduces an additional subset-specific temporal loss without constructing additional temporal jigsaw puzzles.

**Figure A2:**
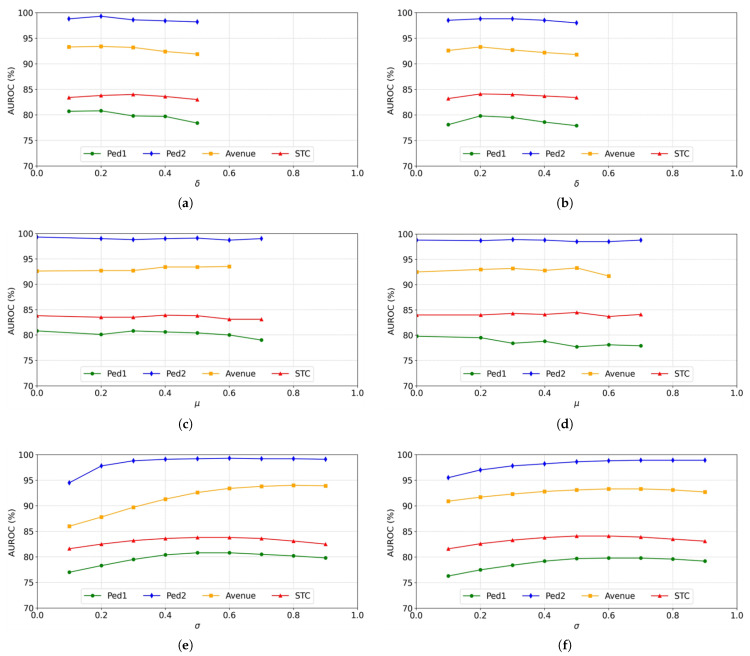
Parameter sensitivity analysis for thresholds δ and μ, and weight σ. The performance is measured by micro-averaged AUROC (%). (**a**) Threshold δ (CTJiP); (**b**) Threshold δ (ETJiP); (**c**) Threshold μ (CTJiP); (**d**) Threshold μ (ETJiP); (**e**) Weight σ (CTJiP); (**f**) Weight σ (ETJiP).

## Data Availability

Data will be made available from the corresponding author on reasonable request.

## Appendix A. Gradient-Flow Interpretation of the SETJiP Framework

Figure A1 illustrates the gradient-flow view of DSTJiP and the two training schemes of SETJiP. The notations used in this appendix are summarized in Table A1. This appendix supplements the main text by providing a gradient-flow interpretation of why DSTJiP does not explicitly prioritize global-motion examples in temporal learning and how CTJiP and ETJiP strengthen such temporal prioritization in different ways.

**Table A1:** Summary of notations used in Figure A1.

Notation	Description
*C*	Set of object-centric spatio-temporal cube examples in a mini-batch.
$X_{1},X_{3}$	mini-batch inputs: $X_{1}$ contains both SJiP and TJiP examples,
	while $X_{3}$ contains additional TJiP examples constructed for CTJiP.
$W_{1},W_{2},W_{3}$	Trainable parameters for the feature extractor, spatial classifier,
	and temporal classifier, respectively.
$h^{\left(1\right)},h^{\left(3\right)}$	Feature representations extracted from $X_{1}$ and $X_{3}$, respectively.
$Z^{\left(1\right)}$	Spatial inference matrix produced from $h^{\left(1\right)}$ by the spatial classifier.
$Z^{\left(2\right)}$	Temporal inference matrix produced from $h^{\left(1\right)}$ by the temporal classifier.
$Z^{\left(3\right)}$	Additional temporal inference matrix produced from $h^{\left(3\right)}$ for CTJiP.
$M_{0}$	Selection matrix for motion-based example filtering.
$M_{1},M_{2}$	Matrices for selecting the spatial outputs corresponding to SJiP examples
	and the temporal outputs corresponding to TJiP examples, respectively.
$M_{4}$	Selection matrix for temporal inference matrices corresponding to
	TJiP generated from selected global-motion examples.
$Z_{1},Z_{2},Z_{3},Z_{4}$	Selected outputs for loss calculations.
$L_{s},L_{t},L_{ct},L_{et}$	Loss functions for SJiP, TJiP, CTJiP, and ETJiP, respectively.
β,η	Hyperparameters weighting the all-sample temporal loss and the
	extra subset-specific temporal loss, respectively.
$L,L_{x}$	Total loss and the extra subset-specific temporal loss, where
	$L_{x}$ denotes either $L_{ct}\left(Z_{3}\right)$ or $L_{et}\left(Z_{4}\right)$.

### Appendix A.1. DSTJiP Does Not Explicitly Prioritize Global-Motion Examples

**Training framework of DSTJiP.** As shown by the blue arrows in Figure A1, DSTJiP jointly optimizes SJiP and TJiP using a shared feature extractor. Let $X_{1}$ denote the mixed mini-batch containing both SJiP and TJiP examples. The extracted representation $h^{\left(1\right)}$ is fed into both the spatial and temporal classifiers, producing the spatial inference matrix $Z^{\left(1\right)}$ and the temporal inference matrix $Z^{\left(2\right)}$, respectively. DSTJiP then decouples the two tasks by selecting only the spatial outputs corresponding to SJiP examples and only the temporal outputs corresponding to TJiP examples, i.e., $Z_{1}\;=\;M_{1}Z^{\left(1\right)}$ and $Z_{2}\;=\;M_{2}Z^{\left(2\right)}$. The feature extractor parameters are thus updated by the aggregated gradients:

(A1) $$\begin{array}{c} \frac{\partial L_{s}\left(Z_{1}\right)}{\partial W_{1}}\;+\;\frac{\partial L_{t}\left(Z_{2}\right)}{\partial W_{1}}=\left(\frac{\partial L_{s}\left(Z_{1}\right)}{\partial Z_{1}}\cdot \frac{\partial Z_{1}}{\partial h^{\left(1\right)}}\;+\;\frac{\partial L_{t}\left(Z_{2}\right)}{\partial Z_{2}}\cdot \frac{\partial Z_{2}}{\partial h^{\left(1\right)}}\right)\cdot \frac{\partial h^{\left(1\right)}}{\partial W_{1}}.\; \end{array}$$

Equation (A1) shows that both the spatial and temporal losses backpropagate through the identical representation $h^{\left(1\right)}$. Therefore, DSTJiP performs temporal learning under the joint optimization of the spatial and temporal objectives.

However, within DSTJiP, the temporal loss $L_{t}\left(Z_{2}\right)$ is formed from temporally shuffled examples in the mini-batch without example-specific prioritization: training examples are uniformly sampled, and temporal jigsaw puzzles are assigned equal weights in the temporal loss. As a result, DSTJiP provides no explicit mechanism to prioritize global-motion examples in temporal learning.

Consequently, from a gradient-flow interpretation, the temporal influence of global-motion examples is determined implicitly by their frequency in the mini-batch and by the joint optimization of spatial and temporal objectives.

### Appendix A.2. CTJiP: Strong Enhancement Through Additional TJiP Instances and an Additional Temporal Loss

SETJiP increases the temporal gradient contribution of global-motion examples in two different ways, and CTJiP is the stronger one. As shown by the orange arrows in Figure A1, CTJiP constructs additional temporal jigsaw puzzles only from selected global-motion examples. These additional TJiP examples are fed into the same feature extractor and the same temporal classifier, producing an additional temporal loss computed from the corresponding inference matrices. The gradient of this complementary temporal loss with respect to the feature extractor is

(A2) $$\frac{\partial L_{ct}\left(Z_{3}\right)}{\partial W_{1}}=\frac{\partial L_{ct}\left(Z_{3}\right)}{\partial Z_{3}}\cdot \frac{\partial Z_{3}}{\partial h^{\left(3\right)}}\cdot \frac{\partial h^{\left(3\right)}}{\partial W_{1}}.$$

Equation (A2) shows that CTJiP assigns an additional temporal loss term to selected global-motion examples. When combined with Equation (A1), this means that these examples contribute not only through the original all-sample temporal loss in DSTJiP, but also through an additional subset-specific temporal loss term. Therefore, CTJiP can be interpreted as explicitly and directly increasing the temporal gradient contribution of global-motion examples. Importantly, CTJiP does not strengthen temporal prioritization only through an additional loss term. By constructing additional temporal jigsaw puzzles only from selected global-motion examples, it densifies the supervision on these examples.

Thus, CTJiP provides a strong enhancement of the temporal gradient contribution of global-motion examples, because it combines an additional subset-specific temporal loss with enlarged temporal exposure. Here, temporal exposure refers to how frequently these examples contribute to the temporal objective within each mini-batch.

### Appendix A.3. ETJiP: Conservative Enhancement Through an Additional Subset-Specific Temporal Loss Under a Fixed Total Temporal-Loss Scale

As shown by the green arrows in Figure A1, ETJiP reuses the original temporal jigsaw puzzles in $X_{1}$ and introduces an additional subset-specific temporal loss only for the subset corresponding to selected global-motion examples. Let $Z_{4}\;=\;M_{4}Z_{2}$ denote the selected temporal inference matrices. The gradient of the emphasized temporal loss with respect to the feature extractor is

(A3) $$\frac{\partial L_{et}\left(Z_{4}\right)}{\partial W_{1}}=\frac{\partial L_{et}\left(Z_{4}\right)}{\partial Z_{4}}\cdot \frac{\partial Z_{4}}{\partial h^{\left(1\right)}}\cdot \frac{\partial h^{\left(1\right)}}{\partial W_{1}}.$$

By combining Equations (A1) and (A3), ETJiP introduces an additional subset-specific temporal loss term for selected global-motion examples while reusing the original set of TJiP instances. Unlike CTJiP, it does not increase temporal training exposure by constructing additional temporal jigsaw puzzles. Instead, ETJiP redistributes the original temporal-loss strength between the all-sample temporal loss and the additional subset-specific temporal loss. As a result, ETJiP can be interpreted as increasing the relative temporal gradient contribution of selected global-motion examples while keeping the overall temporal-loss scale unchanged.

ETJiP emphasizes these examples more conservatively by introducing an additional subset-specific temporal loss under a fixed overall temporal-loss scale, thereby keeping the enhancement bounded. In this way, ETJiP can be viewed as increasing the relative share of temporal optimization devoted to selected global-motion examples, rather than enlarging their temporal exposure or further increasing the total temporal-loss scale.

### Appendix A.4. Unified Parameter-Update View

Finally, SETJiP integrates the base DSTJiP optimization with either CTJiP or ETJiP. Following the loss formulation in the main text, the total loss can be written as

(A4) $$L\;=\;L_{s}\left(Z_{1}\right)\;+\;\beta L_{t}\left(Z_{2}\right)\;+\;\eta L_{x}\left(Z_{x}\right),$$

where $L_{x}\left(Z_{x}\right)$ denotes either $L_{ct}\left(Z_{3}\right)$ for CTJiP or $L_{et}\left(Z_{4}\right)$ for ETJiP.

Accordingly, the parameter update of the feature extractor can be written as

(A5) $$\frac{\partial L}{\partial W_{1}}=\frac{\partial L_{s}\left(Z_{1}\right)}{\partial W_{1}}\;+\;\beta \frac{\partial L_{t}\left(Z_{2}\right)}{\partial W_{1}}\;+\;\eta \frac{\partial L_{x}\left(Z_{x}\right)}{\partial W_{1}}.$$

For SETJiP(CTJiP), Equation (A5) becomes

(A6) $$\frac{\partial L}{\partial W_{1}}=\frac{\partial L_{s}\left(Z_{1}\right)}{\partial W_{1}}\;+\;\beta \frac{\partial L_{t}\left(Z_{2}\right)}{\partial W_{1}}\;+\;\eta \frac{\partial L_{ct}\left(Z_{3}\right)}{\partial W_{1}},$$

where CTJiP provides a strong enhancement because selected global-motion examples contribute to both the all-sample temporal loss and the additional subset-specific temporal loss. In addition, CTJiP enlarges the temporal exposure of global-motion examples through additional TJiP instances.

For SETJiP(ETJiP), Equation (A5) becomes

(A7) $$\frac{\partial L}{\partial W_{1}}=\frac{\partial L_{s}\left(Z_{1}\right)}{\partial W_{1}}\;+\;\beta \frac{\partial L_{t}\left(Z_{2}\right)}{\partial W_{1}}\;+\;\eta \frac{\partial L_{et}\left(Z_{4}\right)}{\partial W_{1}},$$

where ETJiP provides a conservative enhancement because it introduces an additional subset-specific temporal loss on selected global-motion examples while keeping the total temporal-loss scale unchanged, i.e., β + η = 1. Thus, ETJiP can be interpreted as increasing the relative temporal gradient contribution of these examples through bounded reallocation between the all-sample temporal loss and the subset-specific temporal loss.

### Appendix A.5. Pseudocodes of Subroutines

This subsection provides the pseudocode of the subroutines used by the training and inference algorithms described in the main text. Their role is to make explicit the implementation-level procedures for data preprocessing, jigsaw-puzzle construction, and subset selection, rather than to introduce additional methodological components.

Specifically, Algorithm A1 builds object-centric spatio-temporal cubes offline by detecting objects in the center frame of each clip and reusing the same bounding box to crop all *l* frames. Algorithm A2 constructs SJiP by applying a uniformly sampled spatial permutation to each patch within a cube, while Algorithm A3 constructs TJiP by temporally shuffling patches within a cube. For SETJiP, Algorithm A4 constructs the additional TJiP instances used by CTJiP from selected global-motion cubes, and Algorithm A5 selects the subset of original TJiP instances emphasized by ETJiP.

**Algorithm A1 **Subroutine: BuildAllCubes (D,l,H,W)
Require: Video set $D\;=\;\{V_{k}|k\;=\;1,\ldots ,K\}$, $V_{k}\;=\;\left\{I_{i}^{k}|i\;=\;1,\ldots ,T^{k}\right\}$; cube length *l* (odd); resized patch size (H×W) Ensure: OCST cube set $C_{\mathrm{all}}$ // These procedures, same as DSTJiP, are precomputed offline. 1: $C_{\mathrm{all}}\leftarrow \emptyset$ 2: **for** each video $V_{k}\in D$ **do** 3: **for** $i\;=\;\frac{l\;+\;1}{2},\ldots ,T^{k}-\frac{l-1}{2}$ **do** 4: // Valid center frames $i\;=\;\frac{l\;+\;1}{2},\ldots ,T^{k}-\frac{l-1}{2}$ for constructing cubes. 5: // Each center frame $I_{i}^{k}$ corresponds to the *l*-frame clip $v_{i}^{k}\leftarrow \left[I_{i-\frac{l-1}{2}}^{k},\ldots ,I_{i+\frac{l-1}{2}}^{k}\right]$. 6: ${\left\{b_{i,m}\right\}}_{m\;=\;1}^{M_{i}}\leftarrow \mathrm{DETECTOBJECTS}\left(I_{i}^{k}\right)$ // Obtain object bounding box $b_{i,m}$ on center frame $I_{i}^{k}$. // If $M_{i}\;=\;0$, no cube will be generated for this center frame. 7: **for** $m\;=\;1,\ldots ,M_{i}$ **do** 8: **for** t = 1,…,l **do** 9: $I\leftarrow I_{\;i-\frac{l-1}{2}\;+\;t-1}^{k}$ // *i*: global frame index; *t*: within-clip index // *t*-th frame in the *l*-frame clip $v_{i}^{k}$ centered at $I_{i}^{k}$ 10: $u_{t}\leftarrow \mathrm{CROP}rop\left(I,b_{i,m}\right)$ // Reuse $b_{i,m}$ to crop all *l* frames. // Crop *I* using the bounding box $b_{i,m}$ to obtain the patch $u_{t}$. 11: $u_{t}\leftarrow \mathrm{RESIZE}esize\left(u_{t},H,W\right)$ // Resize a patch to fixed H×W. // Store as the *t*-th patch $u_{t}$ 12: **end for** 13: $c_{i,m}^{k}\leftarrow \left[u_{1},\ldots ,u_{l}\right]$ // Assemble all patches to construct the cube $c_{i,m}^{k}$. 14: $C_{\mathrm{all}}\leftarrow C_{\mathrm{all}}\cup \left\{c_{i,m}^{k}\right\}$ 15: **end for** 16: **end for** 17: **end for** 18: **return** $C_{\mathrm{all}}$

**Algorithm A2 **Subroutine: ConstructSJiP $\left(C_{s},l,n\right)$
Require: Cube set $C_{s}$; cube length *l*; grid size *n* Ensure: Spatial jigsaw puzzles $Q_{s}$ 1: $Q_{s}\leftarrow \emptyset$ 2: $P_{s}\leftarrow \left\{P_{s}^{1},P_{s}^{2},\ldots ,P_{s}^{(n^{2})!}\right\}$ //$P_{s}$: permutation set for spatial shuffle // $P_{s}^{j}$ denotes the *j*-th spatial permutation in $P_{s}$. // For each cube, uniformly sample $j\in \{1,\ldots ,\left(n^{2}\right)!\}$. // $P_{s}^{j}$: a candidate permutation for spatial shuffle. 3: **for** each cube $c\in C_{s}$ **do** 4: Sample a random permutation index $j\leftarrow U_{\mathrm{int}}\left[1,\left(n^{2}\right)!\right]$ 5: // Apply the same spatial permutation $P_{s}^{j}$ to all patches in this cube. 6: **for** t = 1,…,l **do** 7: Split patch $u_{t}$ in cube c into an n×n grid of mini-patches 8: Reorder the n×n mini-patches by $P_{s}^{j}$ and obtain the shuffled patch ${\tilde{u}}_{t}$ 9: **end for** 10: Generate puzzle $p_{s}\leftarrow \left[{\tilde{u}}_{1},\ldots ,{\tilde{u}}_{l}\right]$ // Spatial Jigsaw Puzzle $p_{s}$ 11: $Q_{s}\leftarrow Q_{s}\cup \left\{p_{s}\right\}$ 12: **end for** 13: **return** $Q_{s}$

**Algorithm A3 **Subroutine: ConstructTJiP$\left(C_{t},l,\delta \right)$
Require: Cube set $C_{t}$; cube length *l*; threshold δ Ensure: Temporal jigsaw puzzles $Q_{t}$ 1: $Q_{t}\leftarrow \emptyset$ 2: $P_{t}\leftarrow \left\{P_{t}^{1},P_{t}^{2},\ldots ,P_{t}^{l!}\right\}$ //$P_{t}$: permutation set for temporal shuffle // $P_{t}^{j}$ denotes the *j*-th temporal permutation in $P_{t}$. // For each cube, uniformly sample j∈{1,…,l!}. // $P_{t}^{j}$: a candidate permutation for temporal shuffle. 3: **for** each cube $c\in C_{t}$ **do** 4: **if** $g_{m}\left(c\right)>\delta$ **then** 5: Sample a random permutation index $j\leftarrow U_{\mathrm{int}}\left[1,l!\right]$ 6: Reorder $\left[u_{1},\ldots ,u_{l}\right]$ by $P_{t}^{j}$ to obtain the shuffled patches $\left[{\tilde{u}}_{1},\ldots ,{\tilde{u}}_{l}\right]$ 7: Generate puzzle $p_{t}\leftarrow \left[{\tilde{u}}_{1},\ldots ,{\tilde{u}}_{l}\right]$ // Temporal Jigsaw Puzzle $p_{t}$ 8: **else** 9: Generate puzzle $p_{t}\leftarrow \left[u_{1},\ldots ,u_{l}\right]$ // Temporal Jigsaw Puzzle $p_{t}$ 10: // Keep original order (still used for training) 11: **end if** 12: $Q_{t}\leftarrow Q_{t}\cup \left\{p_{t}\right\}$ 13: **end for** 14: **return** $Q_{t}$

**Algorithm A4 **Subroutine: ConstructCTJiP $\left(C_{m},l\right)$
Require: Global motion cube set $C_{m}$; cube length *l* Ensure: Complementary temporal jigsaw puzzle set $Q_{ct}$ 1: $Q_{ct}\leftarrow \emptyset$ 2: $P_{t}\leftarrow \left\{P_{t}^{1},P_{t}^{2},\ldots ,P_{t}^{l!}\right\}$ // Candidate temporal permutations 3: **for** each cube $c\in C_{m}$ **do** 4: Sample a random permutation index $j\leftarrow U_{\mathrm{int}}\left[1,l!\right]$ 5: Reorder $\left[u_{1},\ldots ,u_{l}\right]$ by $P_{t}^{j}$ to obtain the shuffled patches $\left[{\tilde{u}}_{1},\ldots ,{\tilde{u}}_{l}\right]$ 6: Generate puzzle $p_{ct}\leftarrow \left[{\tilde{u}}_{1},\ldots ,{\tilde{u}}_{l}\right]$ 7: // Complementary Temporal Jigsaw Puzzle $p_{ct}$ 8: $Q_{ct}\leftarrow Q_{ct}\cup \left\{p_{ct}\right\}$ 9: **end for** 10: **return** $Q_{ct}$

**Algorithm A5 **Subroutine (ETJiP): SelectETJiP $\left(Q_{t},C_{m}\right)$
Require: Temporal jigsaw puzzle set $Q_{t}$; global motion cube set $C_{m}$ Ensure: Emphasized temporal jigsaw puzzle set $Q_{et}$ 1: $Q_{et}\leftarrow \emptyset$ 2: **for** $p_{t}$ in $Q_{t}$ **do** // Select subset of $Q_{t}$ corresponding to $C_{m}$. 3: **if** $O\left(p_{t}\right)\in C_{m}$ **then** // $O(p_{t})$ maps puzzle $p_{t}$ to its originating cube c. 4: $p_{et}\leftarrow p_{t}$ // Denote this $p_{t}$ as $p_{et}$. 5: $Q_{et}\leftarrow Q_{et}\cup \left\{p_{et}\right\}$ 6: // Emphasized Temporal Jigsaw Puzzle $p_{et}$ 7: **end if** 8: **end for** 9: **return** $Q_{et}$

## Appendix B. Additional Experimental Results

### Appendix B.1. Comparison of the Most Relevant Methods

**Temporal View.** Table A2 presents a comparison between SETJiP and its most relevant methods in terms of the temporal view. We found that the temporal view exhibits trends generally consistent with the spatio-temporal view, as shown in Table 2 and Table 3. Comprehensively considering all results, SETJiP (CTJiP) performs the best on Avenue, while SETJiP (ETJiP) performs the best on STC.

**Table A2:** Comparison of the temporal view. Performance is measured by micro-averaged AUROC (%) and AUPRC (%). **Bold** figures: the best score. ★: best results between CTJiP and ETJiP. Underlined figures: the second-best score.

Setting	Method	Micro-Averaged AUROC		AUPRC	
		Avenue	STC	Avenue	STC
VAD	DSTJiP [3]	86.6	80.0	73.7	79.1
	SATJiP [28]	92.8	81.9	78.9	80.1
	SETJiP (CTJiP)	**93.6** ★	81.5	**85.5** ★	79.1
	SETJiP (ETJiP)	92.0	**82.5** ★	83.1	**81.2** ★
Detecting localized abnormal motion	DSTJiP [3]	82.4	85.4	8.9	13.9
	SATJiP [28]	**96.8**	87.4	26.9	14.7
	SETJiP (CTJiP)	93.4	86.0	28.3	15.7
	SETJiP (ETJiP)	94.4 ★	**87.8** ★	**29.4** ★	**20.6** ★

**Incorporating the Masking Scheme**. We further investigate the impact of incorporating the masking scheme [28], as it constitutes one of the key differences between SATJiP and SETJiP. In Table A3, in VAD, we observe that this scheme is beneficial only when adopting CTJiP on STC, while offering no advantage or even leading to performance degradation in other cases. In detecting localized abnormal motion, it benefits both CTJiP and ETJiP on STC but results in either no improvement or even deterioration on Avenue. Although yielding mixed effects across datasets, this masking scheme does not provide consistent performance gains. As a result, this masking scheme is not incorporated in SETJiP, as it may also introduce information irrelevant to VAD despite encouraging a focus on motion-related content.

**Table A3:** Impact of incorporating the masking scheme in CTJiP and ETJiP. Performance is measured by micro-averaged AUROC (%) and AUPRC (%). **Bold** figures: the best score.

(**a**) **VAD**								
**Method**	**Micro-Averaged AUROC**				**AUPRC**			
	**Ped1**	**Ped2**	**Avenue**	**STC**	**Ped1**	**Ped2**	**Avenue**	**STC**
CTJiP + Masking	79.0	98.7	93.2	**84.1**	80.7	99.7	79.0	82.1
CTJiP	**80.8**	**99.3**	**93.4**	83.8	**83.7**	**99.8**	83.5	81.0
ETJiP + Masking	76.7	98.5	91.3	83.5	79.1	99.7	78.9	81.5
ETJiP	79.8	98.8	93.3	**84.1**	81.7	99.7	**84.3**	**82.4**
(**b**) **Detecting localized abnormal motion**								
**Method**	**Micro-Averaged AUROC**				**AUPRC**			
	**Avenue**		**STC**		**Avenue**		**STC**	
CTJiP + Masking	**95.6**		88.6		21.4		17.0	
CTJiP	92.7		86.0		**24.8**		15.7	
ETJiP + Masking	91.7		**89.4**		16.6		**29.5**	
ETJiP	94.6		88.7		21.3		21.6	

### Appendix B.2. Ablation Study

**Impact of components in CTJiP and ETJiP.**Table A4 details the ablation study for CTJiP and ETJiP components. “Base” follows the same training scheme as DSTJiP, jointly learning SJiP and TJiP. “TJiP” indicates the inclusion of an additional TJiP branch introduced in CTJiP, while “δ” and “μ” are the thresholds for the motion-based example filtering. $L_{at}$ denotes an additional temporal loss introduced in ETJiP, which is applied to the filtered training examples. We adopt an incremental design due to the interdependence among the additional TJiP branch, the additional temporal loss, and the example filtering. Specifically, without the additional TJiP branch or temporal loss, motion-based example filtering applied to “Base” completely excludes static or localized-motion examples from temporal learning, which reduces normal-pattern coverage and degrades performance at test time.

**Table A4:** Impact of components in CTJiP and ETJiP. This ablation study incrementally adds components to the Base setting. “TJiP” and “$L_{at}$” denote the additional TJiP branch (CTJiP) and temporal loss (ETJiP), respectively. δ and μ are thresholds for motion-related example filtering. A check mark indicates that the corresponding component is enabled, while an empty entry indicates that it is disabled. Performance is measured by micro-averaged AUROC (%). **Bold** figures: the best score.

(**a**) **CTJiP.**							
**Base**	**TJiP**	δ	μ	**Ped1**	**Ped2**	**Avenue**	**STC**
✓				77.3	98.5	91.9	**83.8**
✓	✓			**81.8**	**99.3**	91.9	83.5
✓	✓	✓		80.8	**99.3**	92.6	**83.8**
✓	✓	✓	✓	79.6	99.1	**93.4**	**83.8**
(**b**) **ETJiP.**							
**Base**	$L_{\mathrm{at}}$	δ	μ	**Ped1**	**Ped2**	**Avenue**	**STC**
✓				77.3	98.5	91.9	83.8
✓	✓			–	–	–	–
✓	✓	✓		**79.8**	**98.8**	92.5	84.0
✓	✓	✓	✓	77.7	98.5	**93.3**	**84.1**

In Table A4a, “TJiP” improves performance on Ped1 and Ped2 but brings almost no improvement on STC. On Ped1 and Ped2, performance improves even without “δ” and “μ” as the abundance of global-motion examples allows temporal learning to focus on them without the example filtering. “δ” improves performance on Avenue, where roughly 20% of the examples in the training set lack motion, by shifting the focus toward motion-related examples. Since less than 50% of training examples in Avenue contain global motion, applying “μ” encourages the model to focus on the temporal learning of these examples, helping mitigate their insufficient temporal prioritization in the temporal objective.

In Table A4b, the results of “Base + $L_{at}$” are omitted, since without motion-based example filtering, $L_{at}$ is applied to the same training examples as the original temporal loss. Thus, under the default setting, i.e., β + η = 1, “Base + $L_{at}$” is equivalent to “Base”. We observe that “δ + μ” outperforms “δ” on Avenue, while leading to lower performance on Ped1. Since over 97% of the examples in STC contain global motion, applying either “δ” or “δ + μ” yields only limited improvement over the “Base” setting.

**Balanced and Temporally Prioritized Learning.** Table A5 compares temporally prioritized learning and balanced spatio-temporal learning. A representative temporally prioritized configuration (β,η) = (1,1) and a representative balanced configuration (β,η) = (0.9,0.1) are used. “$2L_{t}$” represents a naïve version of “ETJiP (Temporally prioritized)” that doubles the weight of the original temporal loss. Similar to Table A4, we investigate the selection of “δ” and “δ + μ”.

For CTJiP, we find that temporal prioritization brings benefits to Ped1 and Ped2 while showing no improvement to Avenue and STC. For ETJiP, we observe that prioritizing temporal learning leads to no improvement on Ped1 and Ped2. In addition, it leads to significant deterioration of STC. Further considering the results of “$2L_{t}$”, these results suggest that prioritizing temporal learning can pose a high risk of overfitting in ETJiP.

**Table A5:** Representative comparison between balanced and temporally prioritized learning. A check mark indicates that the corresponding component is enabled, while an empty entry indicates that it is disabled. Top: CTJiP. Bottom: ETJiP. Performance is measured by micro-averaged AUROC (%). **Bold** figures: the best score within CTJiP and ETJiP.

Method	δ	μ	Ped1	Ped2	Avenue	STC
CTJiP (Balanced)	✓		78.1	98.7	93.0	83.6
	✓	✓	77.7	98.8	93.2	**83.9**
CTJiP (Temporally prioritized)	✓		**80.8**	**99.3**	92.6	83.8
	✓	✓	79.6	99.1	**93.4**	83.8
ETJiP (Balanced)	✓		**79.8**	98.8	92.5	84.0
	✓	✓	77.7	98.5	**93.3**	**84.1**
ETJiP (Temporally prioritized)	✓		77.9	98.6	93.2	81.1
	✓	✓	78.9	98.6	**93.3**	81.5
2$L_{t}$			77.8	**98.9**	91.6	81.4

### Appendix B.3. Parameter Analysis

**Temporal loss weights for CTJiP and ETJiP.** In Table A6, we analyze representative configurations of temporal loss weights. We restrict our analysis to settings where the total weight of the temporal losses is no less than the spatial loss (β + η≥1), covering both balanced and temporally prioritized learning schemes.

For CTJiP, temporally prioritized settings generally yield better performance on Ped1 and Ped2, with the best results achieved at (β,η) = (1,1) on both datasets. On Avenue, the best result is obtained at (1,0.5), while the balanced setting (0.5,0.5) remains highly competitive. In contrast, on STC, temporally prioritized configurations do not show a consistent advantage, as (1,0.1) and (0.9,0.1) achieve the same best performance.

For ETJiP, better performance on Ped1, Ped2, and STC is generally obtained under balanced or weakly prioritized settings, particularly (β,η) = (0.9,0.1) and (1,0.1), whereas stronger temporal prioritization, such as (1,1), is less effective on these datasets. On Avenue, however, the differences across settings are relatively small, with the best performance achieved at (1,1) and (0.9,0.1).

**Table A6:** Impact of temporal loss weights for CTJiP and ETJiP. “Temporal-prioritized” indicates configurations with β + η>1, while “balanced” corresponds to β + η = 1. Performance is measured by micro-averaged AUROC (%). **Bold** figures: the best score.

		Temporal-Prioritized			Balanced	
	(β,η)	(1,1)	(1,0.5)	(1,0.1)	(0.9,0.1)	(0.5,0.5)
CTJiP	Ped1	**80.8**	80.0	79.3	78.1	76.2
	Ped2	**99.3**	98.6	98.3	98.7	98.3
	Avenue	93.4	**94.0**	92.3	93.2	93.9
	STC	83.8	83.4	**83.9**	**83.9**	82.9
ETJiP	Ped1	77.9	77.9	78.8	**79.8**	76.5
	Ped2	98.6	98.5	**99.2**	98.8	98.5
	Avenue	**93.3**	92.8	92.5	**93.3**	93.2
	STC	81.5	83.2	**84.2**	84.1	83.0

**Thresholds δ and μ for motion-based example filtering. **Figure A2a,b show that δ performs best within the range of 0.1 to 0.3, and we therefore focus on the range [0.1, 0.5]. Setting δ too low fails to suppress non-informative motion, while overly large values tend to exclude useful motion examples. Across datasets, δ = 0.2 yields the strongest performance. Notably, while δ was originally used in DSTJiP [3] only to determine temporally shuffled examples, our results indicate that the same parameter with the same setting as in DSTJiP remains effective when extended to example selection, suggesting its robustness.

Figure A2c,d indicate that μ is effective within the range of 0.3 to 0.5. We also test the cases of μ∈{0.0,0.2,0.6,0.7}, but the effective upper limit of μ on Avenue appears to be around 0.6 due to the limited number of global-motion examples. On Avenue, a higher value of μ generally leads to improved performance, with the peak lying around 0.4 to 0.5. On the other datasets, the trends show no consistent peak across different values of μ ranging from 0.2 to 0.6. Overall, performance tends to be reliable when μ is set within the range of 0.3 to 0.5.

**The weight σ for abnormality score. **Figure A2e,f examine the weight σ within the range of 0.1 to 0.9, avoiding 0 or 1 to ensure both spatial and temporal views are used. Performance is relatively low when σ lies between 0.1 and 0.3 and relatively high when σ is set between 0.5 and 0.7. These results suggest that while the spatial view may be less dominant than the temporal view in VAD, it remains essential for the model’s effectiveness.

### Appendix B.4. Limitations

**Motion-based filtering.** SETJiP relies on motion-based example filtering to select global-motion examples. Although the sensitivity analysis shows that the performance is generally reliable within moderate ranges of δ and μ, these thresholds still depend on the motion characteristics of the target dataset. In particular, δ controls the exclusion of static or spurious-motion cubes, while μ controls how strictly global-motion examples are selected. When the motion distribution of a new scene differs substantially from that of the benchmarks, inappropriate thresholds may select too few informative examples or include too many localized-motion examples. Therefore, δ and μ should be regarded as practical hyperparameters rather than universally fixed constants. A possible future direction is to replace fixed thresholding with adaptive motion-aware selection.

**Cross-domain adaptation.** Another limitation is that the present study follows the standard within-benchmark VAD evaluation protocol and does not explicitly address cross-domain adaptation. In this paper, cross-domain adaptation refers to improving robustness when a model trained or tuned under one surveillance distribution is applied to a different target distribution, which may differ in cameras, scenes, viewpoints, illumination conditions, backgrounds, object scales, or normal motion patterns. Recent studies in other visual inspection tasks, such as unsupervised domain adaptation for crack segmentation [65,66], suggest that domain adaptation can improve robustness under distribution shifts. In crack segmentation, such shifts may arise from different image styles, materials, or acquisition conditions. Although these methods target image-level crack segmentation rather than video anomaly detection, they indicate a promising future direction for adapting SETJiP to new surveillance scenes with different appearances and motion characteristics.
